# Genome-wide analysis of the H3K27me3 epigenome and transcriptome in *Brassica rapa*

**DOI:** 10.1093/gigascience/giz147

**Published:** 2019-12-04

**Authors:** Miriam Payá-Milans, Laura Poza-Viejo, Patxi San Martín-Uriz, David Lara-Astiaso, Mark D Wilkinson, Pedro Crevillén

**Affiliations:** 1 Centro de Biotecnología y Genómica de Plantas (CBGP), Universidad Politécnica de Madrid (UPM) - Instituto Nacional de Investigación y Tecnología Agraria y Alimentaria (INIA), Campus de Montegancedo, 28223, Pozuelo de Alarcón (Madrid), Spain; 2 Centro de Investigación Médica Aplicada (CIMA), Universidad de Navarra, Avenida Pío XII 55, 31008, Pamplona, Spain

## Abstract

**Background:**

Genome-wide maps of histone modifications have been obtained for several plant species. However, most studies focus on model systems and do not enforce FAIR data management principles. Here we study the H3K27me3 epigenome and associated transcriptome of *Brassica rapa*, an important vegetable cultivated worldwide.

**Findings:**

We performed H3K27me3 chromatin immunoprecipitation followed by high-throughput sequencing and transcriptomic analysis by 3′-end RNA sequencing from *B. rapa* leaves and inflorescences. To analyze these data we developed a Reproducible Epigenomic Analysis pipeline using Galaxy and Jupyter, packaged into Docker images to facilitate transparency and reuse. We found that H3K27me3 covers roughly one-third of all *B. rapa* protein-coding genes and its presence correlates with low transcript levels. The comparative analysis between leaves and inflorescences suggested that the expression of various floral regulatory genes during development depends on H3K27me3. To demonstrate the importance of H3K27me3 for *B. rapa* development, we characterized a mutant line deficient in the H3K27 methyltransferase activity. We found that *braA.clf* mutant plants presented pleiotropic alterations, e.g., curly leaves due to increased expression and reduced H3K27me3 levels at AGAMOUS-like loci.

**Conclusions:**

We characterized the epigenetic mark H3K27me3 at genome-wide levels and provide genetic evidence for its relevance in *B. rapa* development. Our work reveals the epigenomic landscape of H3K27me3 in *B. rapa* and provides novel genomics datasets and bioinformatics analytical resources. We anticipate that this work will lead the way to further epigenomic studies in the complex genome of *Brassica* crops.

## Background

The epigenome comprises alternative chromatin states that can affect gene activity [1]. These include DNA methylation, the incorporation of histone variants, and the post-transcriptional modification of histones—such as acetylation or methylation on residues in the histone tails, which can ultimately modify the interaction with DNA. Epigenetic marks accumulate in response to internal and environmental cues and persist through mitosis during the lifespan of the organism. A remarkable finding is the extent of the evolutionary conservation in key regulators and mechanisms across the plant and animal kingdoms, suggesting that a very ancient mechanism underlies this epigenetic regulation [2].

The trimethylation of histone H3 lysine 27 (H3K27me3) [2–4] is one of the best examples of epigenetic regulation of the gene expression programs. H3K27me3 generally anticorrelates with gene repression and marks the so-called facultative heterochromatin, a fraction of the genome where gene expression is repressed but can be activated in response to developmental or environmental signals [5]. This epigenetic mark is deposited at target genes by specific histone methyltransferases as part of the Polycomb repressive complex 2 (PRC2). The PRC2 complex, which is conserved from animals to plants, comprises a set of core components and several accessory subunits [5, 6]. In the model plant *Arabidopsis thaliana* (hereinafter referred to as Arabidopsis) the core PRC2 subunits are well conserved and H3K27me3 is crucial for plant development [3]. During plant growth, different sets of genes are expressed in different organs or tissues and the PRC2 complex is needed to maintain these gene expression patterns [5]. The exact mechanism through which H3K27me3 represses gene expression is not fully understood [5, 6], but H3K27me3 is considered a hallmark of gene repression because it is tightly associated with gene silencing.

In plants, H3K27me3 is crucial for developmental transitions such as gametophyte formation, seed germination, and floral initiation [3, 4]. In Arabidopsis, ≥20% of protein-coding genes are covered by H3K27me3 in a given organ [7, 8]; and similar results have been obtained in rice, maize, and the model cereal *Brachypodium distachyon*, suggesting a conserved role of this epigenetic mark in plant development [9–11]. The importance of this histone modification is highlighted by recent reports showing that up to 60% of protein-coding genes are silenced by H3K27me3 in several specific plant cell types [12].

The Brassicaceae or Cruciferae family includes Arabidopsis and several important crops. The *Brassica* genus includes a number of condiments and vegetables, as well as economically important oilseed crops. Brassica crops have complex genomes that underwent a whole-genome triplication with subsequent genome rearrangements and chromosome reduction after the divergence from a common ancestor with Arabidopsis ∼15 million years ago [13, 14]. Therefore, the mesohexaploid Brassica genomes such as *Brassica rapa* (turnip, field mustard; genome AA), *Brassica nigra* (black mustard; genome BB), and *Brassica oleracea* (cabbage; genome CC) are predicted to encode up to 3 orthologs of each Arabidopsis gene. Within the *Brassica* genus, the diploid *B. rapa* is considered a model for genomic studies because it has a small genome size that makes up half of the genomes of the allotetraploids *Brassica juncea* (indian mustard; AABB) and *Brassica napus* (rapeseed; AACC), which are relevant oilseed crops worldwide. *B. rapa* displays an extreme morphological diversity and includes economically important vegetables and oilseed crops [15]. In addition, the *B. rapa* (Chinese cabbage, Chiifu-401) genome has been fully sequenced and annotated and hundreds of accessions have been re-sequenced [14, 15, 16].

Despite intense epigenetic research in Arabidopsis, genome-wide epigenomic studies in *Brassica* crops are scarce. We are interested in understanding the epigenetic regulation underlying key agronomic traits in Brassicaceae. To begin answering this question, here we study the genome-wide levels of H3K27me3 in leaves and inflorescences of *B. rapa* R-o-18, an oilseed variety. We found that H3K27me3 is associated with gene silencing and decorates >25% of *B. rapa* protein-coding genes. Comparative analyses between leaves and inflorescences show that the expression of a number of floral regulators correlates with dynamic changes in H3K27me3. Phenotypic characterization of a mutant in the *B. rapa* homolog of the histone methyltransferase CURLY LEAF (BraA.CLF) revealed the importance of H3K27me3 for proper *B. rapa* development. Mutant *braA.clf* plants showed reduced H3K27me3 and high expression levels of floral identity genes in leaves, resulting in a number of pleiotropic developmental alterations. Our work highlights the importance of H3K27me3 deposition in the regulation of developmental transitions in plants and leads the way to further epigenomic studies in *Brassica* crops.

## Data Description

In this work we obtained the genome-wide profile of the epigenetic mark H3K27me3 in *B. rapa*. We performed chromatin immunoprecipitation followed by high-throughput sequencing (ChIP-seq) from leaves and inflorescences. To correlate histone methylation and messenger RNA (mRNA) levels, we extracted RNA from the same harvested material and 3′-end mRNA high-throughput sequencing (3′RNA-seq) was performed. To uncover the relevance of H3K27me3 in *B. rapa* development, we characterized a tilling mutant line deficient in the H3K27 methyltransferase BraA.CLF. ChIP-seq data (225 million paired-end fragments) and 3′RNA-seq data (75 million single-end reads) were archived at NCBI SRA under the accession number PRJNA542357.

## Analyses

### A reproducible epigenomic analysis pipeline

To enhance compliance with the FAIR principles (findability, accessibility, interoperability, and reusability) for scholarly digital objects [17], we designed a Reproducible Epigenomic Analysis (REA) pipeline for ChIP-seq and RNA-seq using Galaxy [18], an open web-based platform where each analytical step is formally documented and can be shared and reproduced. We generated Galaxy workflows for the analysis of both ChIP-seq data and RNA-seq data. These workflows were executed on a locally administered Galaxy server via a Docker container image [19]. The Docker technology allowed us to bundle all Galaxy components and tools into a distributable package, which is publicly available for download and execution. Analytical steps that could not be integrated within a Galaxy workflow were captured and documented in Jupyter notebooks [20], an open-source interactive computing environment that allows sharing of code, documentation, and results. These notebooks are also available within a Docker image that runs Jupyter. The REA pipeline is available in the GitHub repository [21] and in the associated Zenodo release [22].

**Figure 1:**
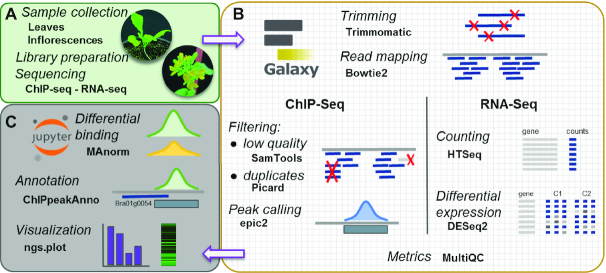
Schematic view of the analytical workflow of the Reproducible Epigenomic Analysis pipeline (REA). (A) Samples from leaves and inflorescences were used for ChIP-seq and RNA-seq. (B) Major analytical steps were conducted in a reproducible Galaxy workflow, running on a Docker container. (C) Further analysis and graphical representation of results were tracked and run on Jupyter interactive notebooks.

In Fig. 1, a schematic representation of our REA pipeline is shown. We used well-established tools including Bowtie2 [23] for short-read sequence alignment, HTSeq [24] for feature mapping quantification, epic2 [25] for ChIP-seq peak calling, MAnorm [26] for quantitative comparison of ChIP-seq data, and DESeq2 [27] for differential gene expression analysis. A detailed description of these workflows can be found in the Methods section.

### Genome-wide identification of H3K27me3 regions in *B. rapa*

To study the epigenetic landscape of H3K27me3 in *B. rapa* we performed ChIP experiments from leaf and inflorescence samples. Immunoprecipitated chromatin and Input (chromatin extracts not subjected to immunoprecipitation) DNA libraries were sequenced by Illumina technology at 125-bp paired-end reads; >30 million fragments were obtained for each sample (Table S1) and sequencing data were analyzed using the REA pipeline. Raw paired-end reads were trimmed to remove low-quality bases and short reads, with >99% of ∼123 bp-long reads being maintained. Two of the most commonly used aligners for ChIP-seq analysis are Burrows-Wheeler Aligner (BWA) [28] and Bowtie2, which carry out fast mapping of DNA sequences using the Burrows-Wheeler transform method. We tested which of these aligners best suited our samples using the latest *B. rapa* v3.0 Chiifu-401 genome as reference [14, 16]. Although BWA yielded comparable results, we obtained a small increase on paired-end mapping efficiency using Bowtie2 (Table S2), and this algorithm was therefore used for the remainder of our genomic analyses in *B. rapa*. Mapping with Bowtie2 against the *B. rapa* v3.0 genome [16] yielded an average 82% mapping rate, where 42–61% of the reads mapped to multiple locations (Table S1), likely reflecting the mesopolyploid nature of the *B. rapa* genome or the abundance of repeated DNA elements. After mapping, duplicated reads were removed and ChIP-seq signal distribution over *B. rapa* genome was visualized and inspected using the Integrative Genomics Viewer (IGV) [29].

**Figure 2:**
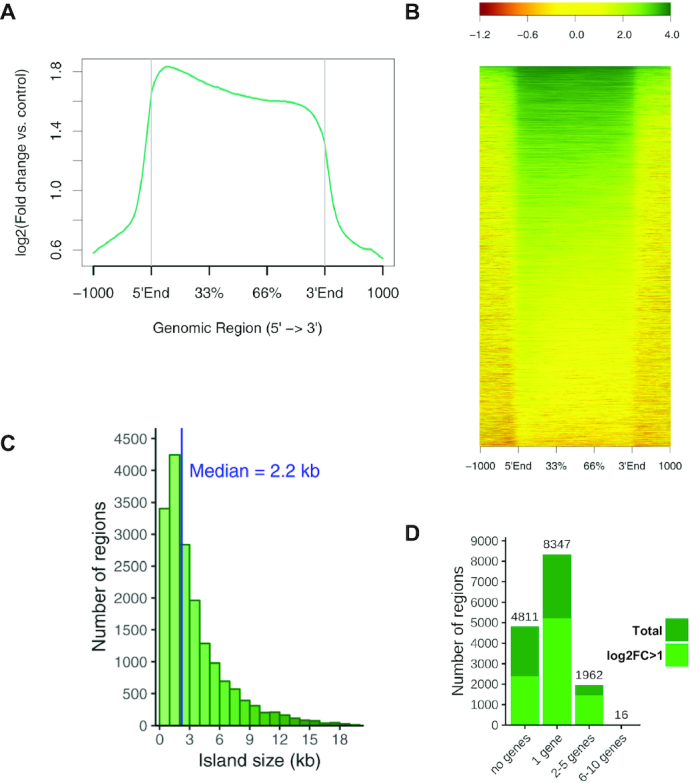
ChIP-seq analysis of H3K27me3 regions in *B. rapa* leaves. (A) Metagene plot with the average profile of mean ChIP-seq signal over marked genes. (B) Gene heat map of H3K27me3 marked genes, scaling genes to a same size flanked by 1-kb regions. Color scale indicates the log_2_ ratios of ChIP samples compared to input. (C and D) Distribution of H3K27me3 peak regions by length (C) and number of covered genes (D). Total and high-confidence (log_2_FC > 1) H3K27me3 peaks are represented in (D).

Using the REA pipeline, we then determined the overall distribution patterns of H3K27me3 in *B. rapa* leaves. A metagene plot of H3K27me3 ChIP-seq signal showed that, as described in other plant species [7, 9, 10], H3K27me3 is not enriched at promoter regions but rather covers *B. rapa* coding regions, showing little preference for the 5′ versus 3′ ends of genes (Fig. 2A). A heat map representation of the genome-wide ChIP-seq signal showed that H3K27me3 accumulation displays a gradual variation between genes, with some genes showing high histone methylation density and others relatively low H3K27me3 levels (Fig. 2B). To precisely determine the location of H3K27me3-marked regions across the genome, we performed a ChIP-seq peak-calling analysis, adjusting software settings for detection of broad-range peaks. We compared 2 widely used but different peak-calling algorithms: MACS2 [30], an algorithm initially designed to identify sharp peaks but extended to detect broad peaks such as those arising from this analysis; and epic2, a highly performant implementation of SICER [31], an algorithm designed for noisy and diffuse ChIP-seq data such as histone methylation. Both peak-finding algorithms detected a comparable number of peaks (MACS2 20,000 and epic2 15,000 peak regions). However epic2 was able to detect wider histone methylated regions than MACS2 (Fig. S1), with a mean peak size of 2,991 bp for epic2 compared to 1,985 bp for MACS2, and was the preferred tool for studying diffuse epigenetic marks in our study.

Our REA pipeline identified 15,136 H3K27me3 marked regions in *B. rapa* leaves using epic2 (Tables S3 and S4), where 68% overlapped with ≥1 protein-coding genes (Fig. 2C and D, Table S3). We found 12,480 genes marked with H3K27me3, which represents 27% of all *B. rapa* protein-coding genes. In animals H3K27 methylated regions cover hundreds of kilobases and usually span several genes [6, 7]. We found that the average size of *B. rapa* H3K27me3-marked regions was ∼3 kb and that 55% of these peak regions were associated with single genes (Fig. 2C and D, Table S3). This is similar to what has been described for Arabidopsis [7]. However, consistent with the complexity of the *B. rapa* genome, we also found ∼2,000 multigenic regions including some large H3K27 methylated regions spanning >10 kb (Table S4). The same overall conclusions were drawn when H3K27me3 ChIP-seq data from *B. rapa* inflorescences were analyzed (Fig. S2, Tables S3 and S5). In summary, our analysis indicates that H3K27me3 marks a great portion of the *B. rapa* genome but it is mainly associated with single genes.

### H3K27me3 correlates with low transcript levels in *B. rapa*

**Figure 3:**
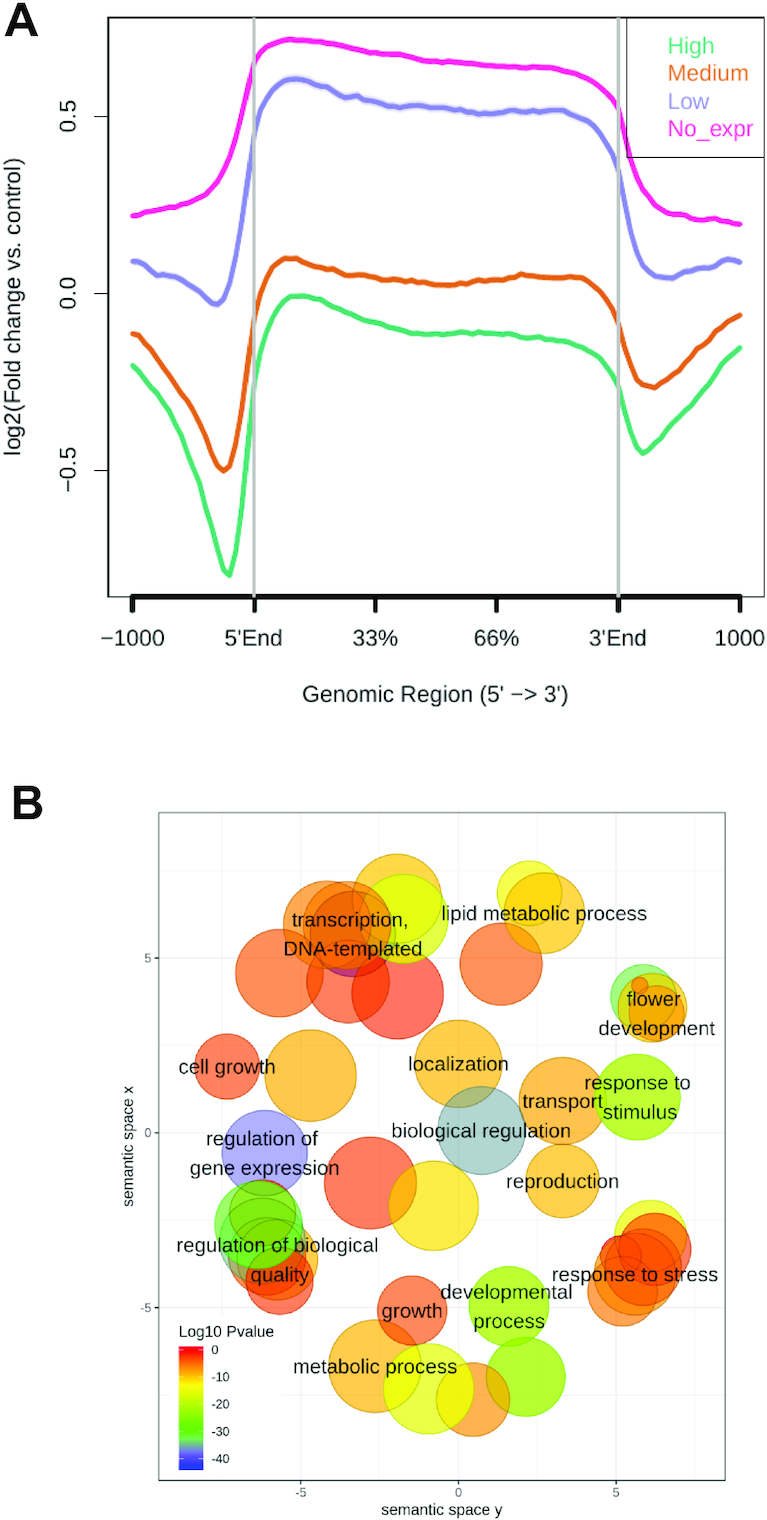
Influence of H3K27me3 on gene function. (A) Metagene plot of H3K27me3 ChIP-seq signal in genes categorized by gene expression level in *B. rapa* leaves. Genes were classified as high, medium, low, and no expression on the basis of their normalized RNA-seq levels. (B) Semantic clustering of enriched GO terms of H3K27me3 marked genes in *B. rapa* leaves. SEA was performed with agriGO (default background = TAIR10_2017 genome locus) and the significant GO terms were clustered with the REVIGO tool. Biological process terms from GO are positioned in a 2-D space derived by multidimensional scaling to a matrix of the semantic similarity of GO terms. The size of the bubble indicates the frequency of the GO term in the underlying Arabidopsis TAIR10 gene ontology and *P*-values are indicated by a color scale.

To identify the correlation between H3K27me3 and gene expression, we performed 3′RNA-seq on the same leaf and inflorescence samples used for ChIP-seq. Profiling RNA levels by 3′RNA-seq and other tag-based methods usually gave higher dynamic detection range and improved mRNA quantification than full transcriptome sequencing [32]. Following the REA pipeline, 3′RNA-seq reads were mapped with Bowtie2 (mapping rate, 77% inflorescences and 86% leaves; Table S1) and counted to obtain quantitative mRNA data from both *B. rapa* organs (Table S6). After normalization of expression data, genes were sorted into 4 categories according to their mRNA levels and used to separately represent their average ChIP-seq H3K27me3 signal on a metagene plot. As shown in Figs   3A and S3A, genes with high levels of H3K27me3 usually exhibit low or no expression, indicating that, as in most eukaryotes, H3K27me3 in *B. rapa* is associated with gene silencing.

epic2 reports regions that pass a score threshold for ChIP-seq read enrichment over input. To select for genes potentially regulated by H3K27me3 in *B. rapa*, we selected high-confidence peak regions where the ChIP signal determined by epic2 was a >2-fold increase over the background (log_2_FC > 1). This analysis identified 8,510 and 7,445 high-confidence H3K27me3-marked genes in leaves and inflorescences, respectively, that showed notable overlap (Fig. S3B). These gene lists were used as input for singular enrichment analysis (SEA) of Gene Ontology (GO) terms using agriGO [33]. The resulting GO term list was summarized and reduced in complexity using REVIGO [34]. The results in Figs 3B and S3C show that in both plant organs, in *B. rapa*, H3K27me3-marked genes are enriched in biological process categories related to metabolism (GO:0019222, GO:0044237), biological regulation (GO:0065007), response to stimulus (GO:0050896), transport/localization (GO:0006810, GO:0051179), development (GO:0032502, GO:0040007), and reproduction (GO:0000003). In addition to response to stimulus, H3K27me3-marked genes in leaves were enriched in response to stress (GO:0006950) and in inflorescences in response to endogenous stimulus (GO:0009719), suggesting that this epigenetic mark plays a role in coordinating genomic responses to either external or internal cues. Genes related to metabolism are marked differently: lipid metabolism genes (GO:0006629) were enriched in leaves, whereas carbohydrate and secondary metabolism GO categories (GO:0008152, GO:0005975, GO:0019748, GO:0009058) were enriched in inflorescences.

In several eukaryotic organisms, H3K27me3-mediated silencing regulates the transition between developmental programs [2, 3]; consistently, genes involved in development and reproduction are significantly marked in both organs. In inflorescences, we found that H3K27me3-marked genes were enriched in GO categories related to cellular/organism processes (GO:0009987, GO:0030154, GO:0051704, GO:0007154). Remarkably, we found that a significant number of genes repressed by H3K27me3 in *B. rapa* leaves were related to transcription (GO:0006351, GO:0010468), cell growth (GO:0016049), and more specifically to flower development (GO:0009908) categories. These data led us to explore how H3K27me3 contributes to the silencing of floral developmental genes in *B. rapa* leaves.

### Dynamic changes in H3K27me3 are associated with the floral transition in *B. rapa*

**Figure 4:**
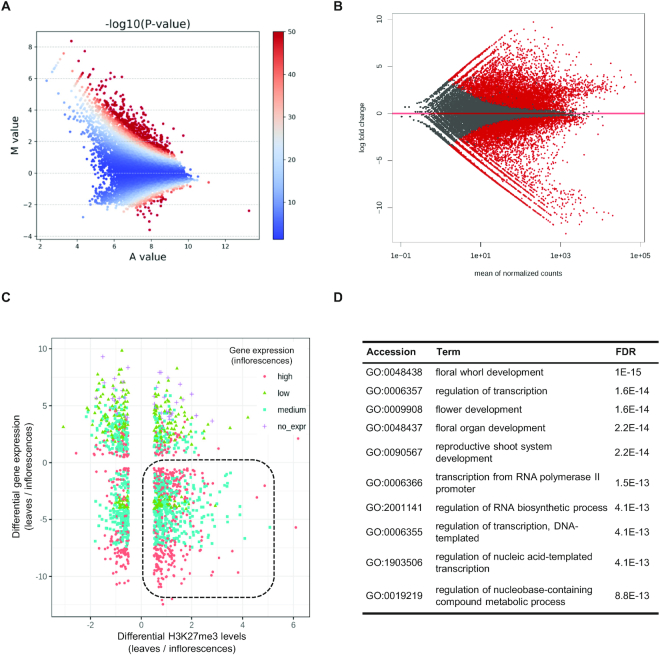
Differential accumulation of H3K27me3 between *B. rapa* leaves and inflorescences. (A) MA plot showing the differential accumulation of H3K27me3 on ChIP-seq peaks determined with MAnorm. Significance is indicated with color scale; red indicates −log_10_(*P*-value) > 50. Normalized read densities of leaves relative to inflorescences were compared to represent the average signal strength of samples (*A* value) against their log_2_ fold change (*M* value) of each peak. (B) MA plot showing the comparison of normalized gene expression between leaves and inflorescences determined with DESeq2; significant DEGs (*P*-adj < 0.1) are colored red. (C) Distribution of genes marked by H3K27me3 in leaves comparing the differential levels of H3K27me3 and mRNA (*P*-adj < 0.1) on leaves vs inflorescences. Dot color indicates mRNA level category (high, medium, low, or no expression) in inflorescences. (D) Top enriched GO terms in the subset of H3K27me3-regulated genes with decreased mark deposition (*M* > 0) and increased gene expression (FC < 0) in inflorescences compared to leaves. Enrichment was determined by SEA on agriGO using all DEGs as custom background. FDR: false discovery rate.

The transition from vegetative to reproductive development is a crucial step in the plant life cycle [35]. This process is highly regulated and involves a genome-wide transcriptional reprogramming where different developmental programs are activated or repressed [8, 12]. To compare H3K27 methylated genes between leaves and inflorescences, we selected the high-confidence peaks from both plant organs and performed a quantitative ChIP-seq signal comparison using MAnorm. MAnorm generates a set of representative peaks after merging overlapping peaks between samples and quantifies their read density; values from both organs were compared on an MA plot and normalized with LOWESS regression. Setting a |*M*| > 0.5 cut-off (where *M* represents the log_2_ fold-change of the normalized read densities in leaves relative to inflorescences), we found 5,986 differentially H3K27me3-marked regions between leaves and inflorescences including 4,729 differentially marked genes (Fig. 4A and Table S7). Thus, ∼55% of all 10,726 merged peaks (from both leaves and inflorescences) showed a significant histone methylation change between these plant organs.

**Figure 5:**
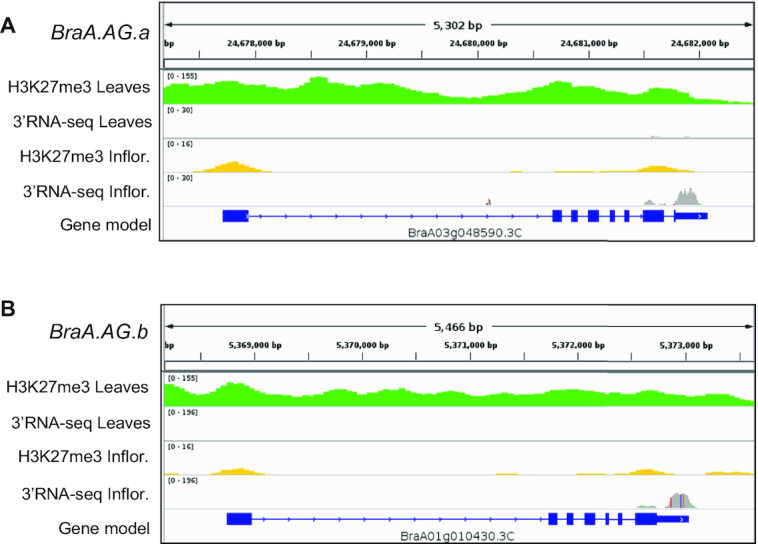
Example of H3K27me3-marked floral regulator loci. IGV viewer snapshots showing the H3K27me3 ChIP-seq and 3′RNA-seq data of *BraA.AG.a* (A) and *BraA.AG.b* (B) in *B. rapa* leaves and inflorescences.

H3K27me3 changes are not always correlated with gene expression changes [2, 6]; e.g., a reduction of H3K27me3 levels may not be enough to promote transcription in the absence of a specific transcription factor. To examine the extent to which the observed H3K27me3 differences in *B. rapa* were correlated with the gene expression changes required for the formation of flowers, differentially expressed genes (DEG) between leaves and inflorescences were determined with our RNA-seq data analysis workflow. Using DESeq2 under our REA pipeline, we found 14,697 DEG (Table S4 and Fig. 4B; |log_2_FC| > 0.5, *P*-adj < 0.1) comparing mRNA levels in leaves relative to inflorescences, indicating that ∼32% of *B. rapa* protein-coding genes are differentially expressed between these organs. To study the correlation between differential H3K27me3 deposition and differential mRNA levels between leaves and inflorescences, we represented the differences in H3K27me3 signal (MAnorm *M* value) against the differential expression levels (DESeq2 log_2_FC) between leaves and inflorescences (Fig. 4C). This revealed 729 loci with reduced H3K27me3 levels (*M*> 0) and increased mRNA expression in inflorescences (DESeq2 log_2_FC < 0) (Fig. 5 and Table S8). Interestingly, SEA analysis of this set of H3K27me3-regulated genes showed that the primary enriched GO terms were related to processes involved in the development of the flower: floral whorl development (GO:0048438), floral organ development (GO:0048437), and flower development (GO:0009908) (Fig. 4D). Very interestingly, this list was enriched in well-known *B. rapa* floral regulators such as *B. rapa FT*-like genes [36] and >60 MADS-box genes. MADS-box proteins are conserved transcription factors that play an important role in the control of flowering time and reproductive development in plants [36]. The list of H3K27-regulated genes in Table S8 includes a number of *B. rapa* MADS-box genes with homology to floral homeotic genes like *AGAMOUS* (Fig. 5), *APETALA1, CAULIFLOWER, PISTILLATA*, and *SEPALLATA* genes, or involved in the floral transition like *AGL19, FRUITFUL*, and *SOC1* [37]. All these data suggest that H3K27me3 is important for the expression of floral regulatory genes and, eventually, the proper development of the reproductive structures in *B. rapa*.

### H3K27me3 deposition is crucial for *B. rapa* development

In Arabidopsis, there are three H3K27me3 methyltransferases: the main one is CURLY LEAF (CLF) [3, 38], which is partially redundant with SWINGER [39], and MEDEA, which is only expressed in the female gametophyte and seeds [40]. CLF mutations result in defective floral morphology, early flowering, and severe leaf developmental alterations [38]; these developmental defects are due to misexpression of H3K27me3-silenced target genes [39, 41].

**Figure 6:**
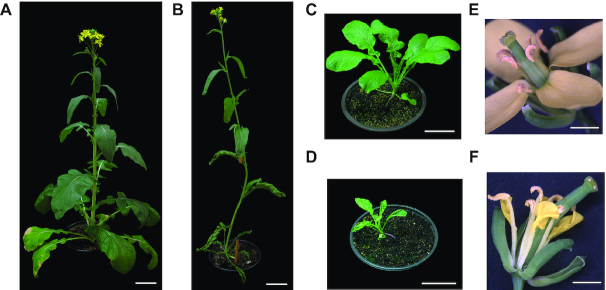
Characterization of *braA.clf-1* mutant. (A and B) Pictures of *B. rapa* R-o-18 wild-type (A) and *braA.clf-1* (B) at flowering stage (bar scale 5 cm). (C and D) Pictures of *B. rapa* R-o-18 wild-type (C) and *braA.clf-1* (D) at 25 DAG (bar scale 5 cm). (E and F) Pictures of *B. rapa* R-o-18 wild-type (E) and *braA.clf-1* (F) flowers (bar scale 1 mm). The *braA.clf-1* mutant shows smaller and curly leaves, and not well expanded flowers with curled sepals and petals.

In the *B. rapa* genome there is one CLF, one SWINGER, and two MEDEA-like homologs [42]. To determine the effect of impaired H3K27 deposition on the development of *B. rapa*, we studied the function of *BraA.CLF* (*BraA04g017190*.3C). Consistent with a possible role as a general histone methyltransferase across the plant, we found that the *BraA.CLF* (*BraA04g017190*.3C) gene is expressed in *B. rapa* leaves and flowers (Fig. S4). We obtained a tilling mutant line [43], *braA.clf-1* (Gln615Stop), that produced a truncated protein without the catalytic domain. Mutant *braA.clf-1* plants have a smaller overall size, reduced expansion of flowers, and curled sepals and petals (Figs 6 and S5). In addition, some mutant flowers showed homeotic transformation of floral organs (Fig. S4C). However, the most striking phenotype of the mutant was the upward curling of leaves, which was most severe on younger leaves (Figs 6D and S5A). The small plant size, altered floral development, and curled leaves resemble the Arabidopsis *clf* mutant [38], suggesting a high degree of functional conservation between both species.

**Figure 7:**
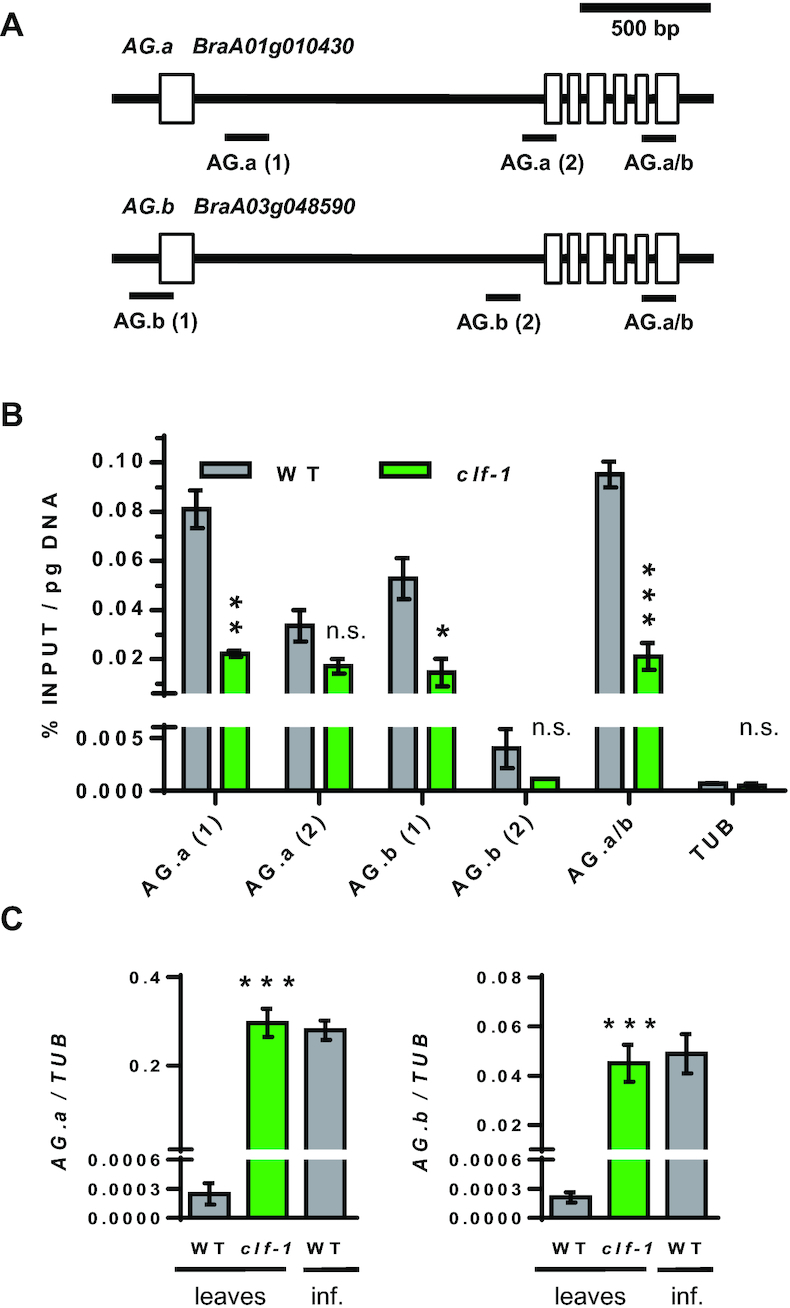
BraA.CLF represses *BraA.AG.a* and *BraA.AG.b* expression in leaves. (A) Cartoon depicting a representation of *BraA.AG.a* and *BraA.AG.b* loci and the chromatin regions analyzed by ChIP-qPCR. (B) ChIP-qPCR showing the H3K27me3 levels at *AG*-like genes in *B. rapa* wild-type vs *braA.clf-1* mutant. Histone modification levels as determined by quantitative qPCR amplification; data represent the average of 2 biological replicates; error bars indicate ±s.d. (n = 2); * *P* < 0.05, ** *P* < 0.01, *** *P* < 0.001 (Student *t*-test). ChIP enrichments were quantified as %INPUT normalized to total DNA content determined by QUBIT assay. As negative control H3K27me3 levels at active housekeeping *gene BraA.TUBULIN* (*BraA10g026070.3C*) are shown in the figure. (C) RT-qPCR data expression levels of *AG*-like genes in *B. rapa* wild-type vs *braA.clf-1* mutant. Data represent the average of 3 biological RNA replicates; error bars indicate ±*s.d*. (n = 3); *** *P* < 0.001 (Student *t*-test, leaves data comparison).

The upward curved leaves in Arabidopsis *clf* mutant are due to the upregulation of the floral identity gene *AGAMOUS (AG)*, which is a direct CLF target in the leaf [38, 44]. In our genomic analysis (Fig. 5 and Table S8), we found that the two *B. rapa AG* loci [45], *BraA01g010430.3C* (*BraA.AG.a*) and *BraA03g048590.3C* (*BraA.AG.b*), showed lower expression and higher H3K27me3 in leaves than inflorescences. Thus, we wondered whether BraA.CLF may be repressing these key floral regulatory genes in *B. rapa* leaves. We performed ChIP experiments and tested discrete chromatin regions by real-time quantitative PCR (ChIP-qPCR) and found that H3K27me3 levels were reduced at *BraA.AG.a* and *BraA.AG.b* loci in mutant *braA.clf-1* leaves (Fig. 7A and B). This reduction in H3K27me3 was associated with increased *BraA.AG.a* and *BraA.AG.b* mRNA levels in *braA.clf-1* mutant leaves as determined by real-time quantitative reverse transcription PCR (RT-qPCR) (Fig. 7C). All these data indicate that BraA.CLF is a major histone methyltransferase regulating the deposition of H3K27me3 at key developmental genes in *B. rapa*; and, more importantly, that the correct H3K27me3 deposition is crucial for the optimal growth of *Brassica* crops.

### Discussion

In this work, we determined the genome-wide and organ-specific distribution of H3K27me3 together with the transcriptome dynamics in leaves and inflorescences of *B. rapa* R-o-18, an oilseed cultivar. We found that H3K27me3 is present in a large number of genomic locations across the *B. rapa* genome, mainly associated with protein-coding genes. Interestingly, SEA analyses showed that H3K27me3-marked genes are enriched in GO terms related to gene regulation, such as “regulation of gene expression” (GO:0010468). In parallel with our ChIP-seq experiments, to correlate histone modification levels with transcript levels, we performed mRNA quantification by 3′RNA-seq. As in other organisms, we found that H3K27me3 is anticorrelated with mRNA levels, indicating that this epigenetic mark is also a hallmark for gene inactivation in *B. rapa*. All these data suggest that H3K27me3 plays an important role in the regulation of gene expression in *Brassica* crops.

Our analysis also revealed that a number of genes related to floral development were marked by H3K27me3 in *B. rapa* leaves. Floral development and the proper formation of the reproductive organs of the plant are crucial for the formation of fruits and seeds, and have a great impact on crop yield. To find out the role of H3K27me3 in floral development in *B. rapa*, we performed a differential analysis of ChIP-seq and 3′RNA-seq signal in leaves vs inflorescences. We found that genes involved in the floral transition or floral organ identity are highly methylated and not expressed in leaves; whereas in inflorescences, the high mRNA levels of this group of genes were associated with a significant decrease of H3K27me3. These data indicate that the repressive H3K27me3 mark is playing a role to prevent ectopic expression of floral regulator genes outside reproductive tissues.

The trimethylation of histone H3K27 is performed by conserved Polycomb PRC2 complexes. In the model plant Arabidopsis, CLF is the main H3K27 methyltransferase. In this work, we isolated a tilling mutant line on the sole *B. rapa* CLF homolog. Phenotypic analysis showed that *braA.clf-1* mutant plants display several developmental defects such as a characteristic curved leaf phenotype. Further ChIP and RT-qPCR experiments showed that *BraA.AG.a* and *BraA.AG.b*, the *B. rapa* homologs of the floral identity gene *AG*, were upregulated due to reduced H3K27me3 in *braA.clf-1* mutant leaves. All these data suggest that BraA.CLF is a bona fide H3K27 methyltransferase and demonstrate the important role of this epigenetic mark for the correct development of *B. rapa* plants.

The *Brassica* genus contains a diverse collection of economically important crop species, including the third-highest produced oil crop in the world and constituting an increasingly popular source of nutrients due to their anticancer, antioxidant, and anti-inflammatory properties and high nutritional value []. The *B. rapa* genome was the first sequenced *Brassica* crop [14], but in recent years the genome sequences of cabbage (*B. oleracea*), black mustard (*B. juncea*), and rapeseed (*B. napus*) have been released [46]. The comprehensive study of H3K27me3 in *B. rapa* presented here opens the door to explore other *Brassica* crops with more complex genomes. We hope that the epigenomic datasets and bioinformatic analytical pipelines generated in this work will aid future studies to shed more light on the epigenetic regulation in plants.

### Potential implications

Here we presented novel H3K27me3 epigenomic and transcriptomic *B. rapa* datasets. To understand the molecular mechanisms of epigenetic regulation in plants is important as fundamental knowledge but also has enormous implications for crop improvement and agriculture [47]. DNA sequence variability alone cannot explain all the diversity of plant phenotypes [48]. The isolation of stable epigenetic variants of several crops has led to the idea that epigenetics could contribute to heritable natural variation that can be selected in plant breeding and crop improvement programs [47, 48].

To facilitate transparency and reproducibility, the bioinformatic pipeline we generated for ChIP-seq and RNA-seq—the Reproducible Epigenomic Analysis pipeline—has been made publicly available, together with the output data being extensively annotated in conformance with the FAIR data principles. The pipeline was constructed using a set of well-established genomic tools and approaches, using a combination of a Galaxy environment and Jupyter notebooks [20]. Both Galaxy and Jupyter provide analytics environments that can be reproduced by anyone through a user-friendly Web interface, providing a platform for others to execute similar analyses following our approach.

## Methods

### Plant material

*B. rapa* yellow sarson (*ssp. trilocularis*) R-o-18 wild-type and *braA.clf-1* (ji32391-a; Gln615Stop) mutant seeds were obtained from RevGenUK [43]. For *braA.clf-1* characterization, we used sibling homozygous mutant plants derived from a backcross to the parental line; all *braA.clf-1* plants were genotyped by PCR and sequencing using the primers in Table S9. Plants were sown in 15 cm diameter plastic pots containing a mixture of substrate and vermiculite (3:1) with added controlled-release fertilizer (Nutricote, Projar Ltd., Spain)) and then grown in controlled-environment plant growth chambers with day/night temperatures of 21/19°C, a mix of cool-white and wide-spectrum FLUORA fluorescent lights (100 µE/m^2^s), and 16 h of light followed by 8 h of darkness. Pictures of flowers were taken using a Leica MZ10 F Stereomicroscope.

### Chromatin Immunoprecipitation and DNA sequencing

ChIP experiments were performed using *B. rapa* R-o-18 wild-type primary leaves collected 14 days after germination (DAG) (Fig. S6A), and inflorescences collected when the first flowers started to open (Fig. S6B and C). Sampling was performed at the end of the light period (zeitgeber time ZT16). Each biological replicate comprises samples from 6 independent plants. ChIP experiments were performed using 2 g of tissue as described at our Plant Chromatin Immunoprecipitation protocol V.2 [49]. We used a specific antibody against H3K27me3 (ref. 07–449, Lot No. 273661, Merck-Millipore, Germany). The primers used for the ChIP-qPCR amplification reactions can be found in Table S9.

ChIP DNA was quantified using an Invitrogen Qubit 3.0 Fluorometer (Thermo Fisher Scientific, USA) and ChIP-seq sequencing libraries were prepared using NEBNext Ultra DNA Library Prep kit (ref. 7370, New England BioLabs, USA) starting from 4 ng of immunoprecipitated DNA. Library quality was determined with a Bioanalyzer instrument (Agilent, USA) and concentration was determined using the KAPA Library Quantification Kit (ref. KK4835, Kapa Biosystems, South Africa). One inflorescence and 2 leaves ChIP-seq biological replicates were sequenced at 2 × 125 bp paired-end reads in an Illumina HiSeq 2500 platform using the SBS v4 kit (Illumina, USA) at the Genomics Units of CNAG-CRG (Barcelona, Spain).

### Gene expression profiling and 3′RNA sequencing

Total RNA was extracted from non-crosslinked aliquots of the same harvested plant tissues used for ChIP. We used EZNA Plant RNA Kit (Omega Bio-tek, USA) following the manufacturer's recommendations. Each biological replicate contained samples from 6 independent plants. RNA integrity was checked by agarose gel electrophoresis and complementary DNA (cDNA) was prepared by reverse transcription of 1 µg of total RNA using “Maxima cDNA Kit with dsDNase” (ref. K1671, Thermo Fisher Scientific, USA) according to the manufacturer's instructions, and quantification was performed by RT-qPCR using the LightCycler 480 and SYBR Green I Master mix (ref. 04707516001, Roche, Switzerland). Gene expression was determined by 2^−ΔΔ^*^CT^* method using *BraA.TUBULIN* (*BraA10g026070.3C*) [50] as housekeeping gene. The primers used for RT-qPCR can be found in Table S9.

Libraries for 3′RNA-seq were prepared using a MARS-seq protocol adapted for bulk RNA-seq [51] with minor modifications. Briefly, RNA quality was determined using a TapeStation 4200 system (Agilent, USA) and 100 ng of total RNA were reverse-transcribed using poly-dT oligos carrying a 7 bp-index. cDNAs were then pooled and subjected to linear amplification via *in vitro* transcription. The resulting amplified RNA was fragmented and dephosphorylated. Ligation of partial Illumina adaptor sequences [51] was followed by a second reverse transcription and full Illumina adaptor sequences were added during a final library amplification. Libraries were quantified using a Qubit 3.0 Fluorometer (Thermo Fisher Scientific, USA) and their size profiles examined in a TapeStation 4200 system (Agilent, USA). Three biological replicates for each organ were sequenced at 1 × 68 bp single-end reads in an Illumina NextSeq 500 (Illumina, USA) at the Advanced Genomics Laboratory of the Centro de Investigación Médica Aplicada (CIMA; Pamplona, Spain).

### The Reproducible Epigenomic Analysis pipeline

Analysis of sequencing data was conducted within environments that either allow the assembly of bioinformatics tools into analytical workflows (Galaxy) or serve to share interactive code-containing documents (Jupyter Notebooks). Jupyter Lab's R and bash kernels were installed using Anaconda3 (Anaconda Software Distribution, 2018). The REA pipeline was implemented as a series of steps distributed within a Docker container [52] that includes all required software dependencies. Any user can deploy an REA instance on demand. To be able to download and use a Dockerized version of Galaxy [53], Docker version 18.09.3 was first installed following the documentation on Docker-CE for Ubuntu. Next, Galaxy version 18.05 was locally installed with the following commands:

mkdir -p ∼/DockerFolders/galaxy_v1
docker run -d -p 8080:80 \
-v ∼/DockerFolders/galaxy_v1/:/export/ \
quay.io/bgruening/galaxy:18.05

This local Galaxy server can be accessed and administered on http://localhost:8080/. Most tools were installed from the Galaxy Tool Shed, with the exception of epic2 v0.0.14 [25], which we manually installed inside the Docker container via an interactive session using Python pip and wrapped as a Galaxy tool. Our wrapped epic2 tool has been successfully integrated into Galaxy and published into the Galaxy Tool Shed [54]. Versions and settings of tools used in Galaxy are noted in the workflows published in our DockerHub images, as well as described below.

To facilitate reproducibility of our data analyses, we built and published two Docker images, which contained all dependencies and software requirements to create a ready-to-run analytical workflow (see Availability of Supporting Data and Materials). The first Docker image contains a Galaxy instance with required tools installed and accessory files to download/index the *B. rapa* reference genome and prepare/run workflows with the analytical steps described below. These workflows are designed to download raw sequencing reads from SRA and export the results locally for further processing. The second Docker image, which runs Jupyter Lab, includes software installations and notebooks with instructions to finalize data analysis and explore results; they include, as a default view, the results published here. These images are designed to operate on a shared local directory for Jupyter to access Galaxy results. Detailed instructions on how to deploy and run each container can be found in our GitHub repository README file.

To enhance our compliance with FAIR publishing requirements, metadata files following schema.org format for both the ChIP-Seq and RNA-Seq experiments are available on GitHub. In addition, metadata descriptors for the ChIP-seq and RNA-seq data submissions in the NCBI SRA are available in ISA-Tab format in the same GitHub repository. All of these files are also available in the associated Zenodo snapshot release [22].

### ChIP-seq analysis

Sequencing data were uploaded to a local Galaxy instance in fastqsanger format. Each sample was independently processed and replicates pooled for peak calling. A first step of trimming was performed with Trimmomatic (v0.36.5) (Trimmomatic, RRID:SCR_011848) [55]. Trimmed reads were mapped to the *B. rapa Chiifu* v3.0 genome using Bowtie2 v2.3.4.2 (Bowtie, RRID:SCR_005476) or BWA v0.7.17.3 (BWA, RRID:SCR_010910), and the results were compared with SAMtools Flagstat [56]. Bowtie2 aligned reads were used in subsequent analyses. BAM files were filtered with SAMtools v1.8 (SAMTOOLS, RRID:SCR_002105) by mapping quality (including concordance of mates) and by duplication state (possible duplicate reads that may arise during library preparation), marked by Picard MarkDuplicates v2.18.2.0 (Picard, RRID:SRC_006525) [57]. The set of deduplicated reads was used for ChIP-seq peak calling on pooled replicates using epic2 v0.0.14 or MACS2 v2.1.1 (MACS2, RRID:SRC_013291) for comparison to one another; the epic2 output was then used for downstream processes. Additional steps in the workflow are aimed at collecting quality metrics using MultiQC [58], as well as producing bigwig files using DeepTools 3.1.2 (Deeptools, RRID:SCR_016366) [59] with the coverage of filtered alignments on bin sizes of 50 bp.

Results from data analysis on Galaxy were downloaded locally for further processing and visualization. The *B. rapa* genome v3.0 contains 10 chromosomes but also thousands of smaller scaffolds, with sequences from either nuclear chromosomes or chloroplast/mitochondria organelles. Thus, prior to analysis of differential H3K27me3 levels between leaves and inflorescences, raw peak-calling results from epic2 were curated via selection of nuclear regions and visual inspection on IGV viewer. Differential levels of H3K27me3 histone mark intensities were computed by comparison of read abundances on our curated list of peaks with MAnorm v1.2.0 (MAnorm, RRID:SCR_010869) [26], which uses MA plot methods to normalize read density levels on provided peaks and calculate *P*-values. This comparison is performed using all available reads, including duplicates, resulting in a list of common peaks with the differences on leaf compared to inflorescences. Peaks from either epic2 or MAnorm were annotated according to overlap of *B. rapa* gene models using ChIPpeakAnno (ChIPpeakAnno, RRID:SCR_012828) [60], and the distribution of ChIP signal over genes was visualized with ngs.plot (ngs.plot, RRID:SCR_011795) [61]. To test replicate samples, read density was obtained over bins of 10 kb of the *B. rapa* genome and normalized using scaling factors. These counts were plotted and sample-sample correlations were calculated using the Spearman method, on scatter plots with density curves (plot instructions as described by Martin P. [62]) (Fig. S7).

### RNA-seq analysis

The bulk transcriptomic analysis was performed using a specific RNA-seq analysis workflow on a local Galaxy instance. Single-end reads were trimmed with Trimmomatic and mapped with Bowtie2 against *B. rapa* v3.0 genome. Before counting, available *B. rapa* v3.0 gene models, which only comprise the coding sequences, were extended by 300 bp at each gene's 3′ untranslated regions and used to quantify aligned reads using HTSeq-count (htseq-count, RRID:SRC_011867) script with stranded and intersection of non-empty sets options. The obtained counts were used for mRNA differential expression analysis with DESeq2 1.18.1 (DESeq2, RRID:SCR_015687) to infer gene expression changes of leaves compared to inflorescences. DESeq2 results were downloaded locally for further analysis outside Galaxy in combination with annotated peaks. To test replicate samples, normalized counts were used to visualize sample-sample correlation and calculate the Spearman correlation (Fig. S8) [62]. Categorization of genes by expression level was achieved after transformation of count data into *z*-scores as follows: for each expressed gene and organ, normalized counts were averaged across replicates, subtracted from the dataset average, and divided by dataset s.d. These *z*-scores were used to define genes with low (*z* < −0.5), medium (−0.5 < *z* < 0.5), and high expression (*z* > 0.5).

### Other bioinformatic analyses

Gene Ontology analysis was performed using agriGO v2.0 (agriGO, RRID:SCR_006989) [33] (Fisher statistical test method; Yekutieli Multi_test adjustment method; *P* < 0.05; and Plant GO slim ontology type); data were visualized reduced in complexity and redundant GO terms using REViGO (REViGO, RRID:SCR_005825) [34] with default parameters (allowed similarity = 0.7; semantic similarity measure = SimRel).

Functional annotation of *B. rapa* v3.0 gene models was obtained from Zhang et al. [16]. Custom annotation of gene models from *B. rapa* genome v3.0 was obtained by blasting coding sequences against *B. rapa* genome v1.5 (E-value cutoff of 0.001) and Arabidopsis (TAIR10 proteins, blastx of *B. rapa* coding sequences with an E-value cutoff of 1e−25).

During our analyses of H3K27me3 levels on *B. rapa* AGAMOUS genes, we found that *BraA.AG.a* (*BraA01g010430.3C*) gene structure annotation was not correct in the recent *B. rapa* genome v3.0. We curated *BraA.AG.a* gene structure using AUGUSTUS (Augustus, RRID:SCR_008417) [63] and Bra013364 (*B. rapa* genome V1.5) gene information at the *B. rapa* database [46].

## Availability of Supporting Source Code and Requirements

- Project name: Epigenomics Workflow on Galaxy and Jupyter
- Project home page: https://github.com/wilkinsonlab/epigenomics_pipeline
- Operating systems: Platform independent
- Programming languages: Python, R, Bash
- Other requirements: none
- License: MIT
- RRID:SCR_017544

## Availability of Supporting Data and Materials

ChIP-seq and RNA-seq data sets supporting the results of this article are available at NCBI SRA under the accession number PRJNA542357. Latest versions of the components of the REA pipeline, and instructions to deploy the Galaxy/Jupyter containers and run the analysis, can be found in the GitHub repository https://github.com/wilkinsonlab/epigenomics_pipeline; this is associated with a Zenodo release to match the configuration used in this publication [22]. The REA pipeline is registered as RRID:SCR_017544 and biotools: Epigenomics_Workflow_on_Galaxy_and_Jupyter at SciCrunch and bio.tools databases, respectively. Compiled Docker images are available at https://hub.docker.com/u/mpaya. All supporting data are available in the *GigaScience* GigaDB database [64].

## Additional Files

Figure S1: Differences between epic2 and MACS2 peak-calling algorithms

Figure S2: ChIP-seq analysis of H3K27me3 regions in *B. rapa* inflorescences

Figure S3: Comparisons of H3K27me3 ChIP-seq signal in *B. rapa* inflorescences

Figure S4: Transcript levels of *B. rapa* H3K27 methyltransferase-coding genes

Figure S5: *BraA.clf-1* mutant phenotypes

Figure S6: Pictures of *B. rapa* plant sampling materials

Figure S7. ChIP-seq sample-sample correlation tests

Figure S8. RNA-seq sample-sample correlation tests

Table S1. Alignment statistics

Table S2. Comparison of Bowtie2 and BWA performance

Table S3. H3K27 trimethylated regions identified in *B. rapa*

Table S4. List of H3K27me3 peaks in *B. rapa* leaves

Table S5. List of H3K27me3 peaks in *B. rapa* inflorescences

Table S6. 3′RNA-seq results

Table S7. List of H3K27me3 differentially marked regions

Table S8. H3K27me3-regulated genes

Table S9. Primer list

giz147_GIGA-D-19-00256_Original_Submission

giz147_GIGA-D-19-00256_Revision_1

giz147_Response_to_Reviewer_Comments_Original_Submission

giz147_Reviewer_1_Report_Original_Submission Diep Ganguly -- 8/28/2019 Reviewed

giz147_Reviewer_1_Report_Revision_1 Diep Ganguly -- 11/7/2019 Reviewed

giz147_Reviewer_2_Report_Original_Submission Jingyu Zhang -- 9/1/2019 Reviewed

giz147_Supplemental_Files

## Abbreviations

3′RNA-seq: 3′-end mRNA high-throughput sequencing; AG: AGAMOUS; bp: base pairs; cDNA: complementary DNA; ChIP: chromatin immunoprecipitation; ChIP-seq: ChIP followed by high-throughput sequencing; ChIP-qPCR: ChIP followed by real-time quantitative PCR; CLF: CURLY LEAF; DAG: days after germination; DEG: differentially expressed genes; FAIR: findability, accessibility, interoperability, and reusability; FC: fold change; FDR: false discovery rate; GO: gene ontology; H3K27me3: histone H3 lysine 27 trimethylation; IGV: integrative genomics viewer; kb: kilobase pairs; LOWESS: Locally Weighted Scatterplot Smoothing; *M*: log_2_ fold-change of the normalized H3K27me3 read densities in leaves relative to inflorescences calculated by MAnorm; MACS: Model-based Analysis of ChIP-Seq; mRNA: messenger RNA; NCBI: National Center for Biotechnology Information; PRC2: Polycomb repressive complex 2; REA: Reproducible Epigenomic Analysis; RNA-seq: RNA sequencing; RT-qPCR: real-time quantitative reverse transcription PCR; s.d: standard deviation; SEA: singular enrichment analysis; SICER: Spatial Clustering for Identification of ChIP-Enriched Regions; SRA: Sequence Read Archive.

## Competing Interests

The authors declare that they have no competing interests.

## Funding

This work was supported by grants RTI2018-097749-B-100, BIO2015–68031-R and RYC-2013–14689 to P.C., and BES-2016–078939 fellowship to L.P.V. from the Spanish Ministerio de Economia y Competitividad (MINECO/FEDER, EU); and by the "Severo Ochoa Program for Centres of Excellence in R&D” from the Agencia Estatal de Investigación of Spain, grant SEV-2016-0672 (2017–2021) to the CBGP. M.P.M. was supported by a Postdoctoral contract associated with the Severo Ochoa Program.

## Authors' Contributions

P.C. conceived the work; M.P.M. performed computational biology analyses; L.P.V. performed initial bioinformatic analysis and all experimental research. P.M.U. and D.L.A. performed 3′RNA-seq library preparation and sequencing. M.W. contributed analytical tools and metadata generation. P.C. and M.P.M. wrote the first draft of the manuscript, which was completed with the assistance of L.P.V. and M.W. All authors approved the final version of the article.
