# Supplementary material for: Genome-wide analysis of the H3K27me3 epigenome and transcriptome in Brassica rapa
Source: Gigascience. 2019 Dec 4;8(12):giz147. doi: 10.1093/gigascience/giz147 (PMC6892454; doi:10.1093/gigascience/giz147)
Supplement: giz147_GIGA-D-19-00256_Revision_1 [file giz147_giga-d-19-00256_revision_1.pdf]

GigaScience

Genome-wide analysis of the H3K27me3 epigenome and transcriptome in Brassica rapa

--Manuscript Draft--

**Manuscript Number:**	GIGA-D-19-00256R1
**Full Title:**	Genome-wide analysis of the H3K27me3 epigenome and transcriptome in Brassica rapa
**Article Type:**	Research
**Funding Information:**	Ministerio de Economía, Industria y Competitividad, Gobierno de España (BIO2015-68031-R) — Dr. Pedro Crevillén<br>Ministerio de Economía, Industria y Competitividad, Gobierno de España (RYC-2013-14689) — Dr. Pedro Crevillén<br>Ministerio de Economía, Industria y Competitividad, Gobierno de España (BES-2016-078939) — Miss Laura Poza-Viejo<br>Ministerio de Economía, Industria y Competitividad, Gobierno de España (SEV-2016-0672) — Not applicable
**Abstract:**	Background<br>Genome-wide maps of histone modifications have been obtained for several plant species. However, most studies focus on model systems and do not enforce FAIR data management principles. Here we study the H3K27me3 epigenome and associated transcriptome of *Brassica rapa*, an important vegetable cultivated world-wide.<br><br>Findings<br>We performed H3K27me3 chromatin immunoprecipitation followed by high-throughput sequencing and transcriptomic analysis by 3'-end RNA sequencing from *B. rapa* leaves and inflorescences. To analyze these data we developed a reproducible epigenomic analysis pipeline using Galaxy and Jupyter, packaged into Docker images to facilitate transparency and reuse. We found that H3K27me3 covers about a third of all *B. rapa* protein-coding genes and its presence correlates with low transcript levels. The comparative analysis between leaves and inflorescences suggested that the expression of various floral regulatory genes during development depends on H3K27me3. To demonstrate the importance of H3K27me3 for *B. rapa* development, we characterized a mutant line deficient in the H3K27 methyltransferase activity. We found that *braA.clf* mutant plants presented pleiotropic alterations, *e. g.* curly leaves due to increased expression and reduced H3K27me3 levels at AGAMOUS-like loci.<br><br>Conclusions<br>We characterized the epigenetic mark H3K27me3 at genome-wide levels and provide genetic evidence for its relevance in *B. rapa* development. Our work reveals the epigenomic landscape of H3K27me3 in *B. rapa* and provide novel genomics datasets and bioinformatics analytical resources. We anticipate that this work will lead the way to further epigenomic studies in the complex genome of Brassica crops.
**Corresponding Author:**	Pedro Crevillén<br>Instituto Nacional de Investigacion y Tecnologia Agraria y Alimentaria<br>Pozuelo de Alarcón, Madrid SPAIN
**Corresponding Author Secondary Information:**	
**Corresponding Author's Institution:**	Instituto Nacional de Investigacion y Tecnologia Agraria y Alimentaria
**Corresponding Author's Secondary Institution:**	
**First Author:**	Miriam Payá-Milans
**First Author Secondary Information:**	

Powered by Editorial Manager® and ProduXion Manager® from Aries Systems Corporation

**Order of Authors:**	Miriam Payá-Milans<br>Laura Poza-Viejo<br>Patxi San Martín-Uriz<br>David Lara-Astiaso<br>Mark D Wilkinson<br>Pedro Crevillén
**Order of Authors Secondary Information:**	
**Response to Reviewers:**	RESPONSE TO REVIEWER COMMENTS:

We are grateful for all the comments of both reviewers. Following reviewer's suggestions some parts of the results and methods has been edited. This has improved the paper although our main conclusions remain the same. In this revised version we added new inflorescences ChIP-seq and RNA-seq analyses (Fig. S2 and S3, and table S3), new IGV snapshots of key developmental genes (Fig. 5), new statistical analyses of ChIP-qPCR data (Fig.7), new FigS5 with more representative mutant plant pictures (Fig. S5) and new correlation analyses between replicate genomic samples (Fig.S7 and S8). As suggested, we also registered our pipeline at SciCrunch and bio.tools databases.

We answered all questions and made some clarifications along the text.

Reviewer #1

The authors characterise the distribution of H3K27me3 across B. rapa leaves and inflorescence tissues, alongside its importance for appropriate development and flowering through gene silencing. Overall, the data appears to be solid providing a resource for the community and extends known relationships into new species. I highly commend the authors on the development and execution of the REA pipeline (aligning with FAIR principles), and hope this helps to establish a new "precedent" for increasing accessibility and rigour in our data & analyses.

RESPONSE: We are glad that reviewer #1 expressed such enthusiasm for the development and execution of our bioinformatic pipeline. We share the same hope for reproducibility and rigour.

I have some concerns/requests, which I think will strengthen the paper prior to publication:

1. While this study characterises the distribution of H3K27me3, it is not exploring anything epigenetic. Instead, this reference to epigenetics is reinforcing the vague use of the term, which is what the authors in ref 1 argue against. I recommend the first sentence should be deleted and all references to epigenetic(s) should instead refer to "histone modification(s)", "chromatin mark(s)" or "epigenome", where the latter is clearly defined.
RESPONSE: We see the point of the reviewer #1; the first sentence of the manuscript was deleted and reference 1 modified accordingly.

2. Could the first use of "BraA.CLF" include the full name (i.e. define the abbreviation).
RESPONSE: The text in the last paragraph of the introduction has been edited.

3. Could the authors please clarify whether "from the same plant samples" (p. 7) means from the same tissues but independent plants or aliquots of the same harvested tissue?
RESPONSE: We took samples for RNA and ChIP from the same harvested plants. The text has been edited in Data Description and Methods sections to clarify this point.

4. P. 8 Please include an appropriate citation for the sentence starting with "A metagene plot of H3K27me3 ... as described in other plant species, ..." for clarity.
RESPONSE: We added the proper citations.

Powered by Editorial Manager® and ProduXion Manager® from Aries Systems Corporation

5. For Fig 2A, can the signal from leaf vs inflorescence tissue be plotted separately? Could some measure of variance (standard deviation or standard error) be included in the plots?
RESPONSE: In the Figure 2, only leaves data was shown as representative for the H3K27me3 mark. Following both reviewers suggestions a new Fig. S2 and S3 including the analysis of the Inflorescence ChIP signal has been added. About Fig. 2A, we redid the metagene plots using ngs.plot including the standard error, but the deviation is so small that it is barely visible in the figure.

6. Fig 2C, can the authors please mark the median. I find this more informative than the mean for this type of data.
RESPONSE: As suggested, mean value has been substituted by median in the new Fig2C.

7. P. 9, I am a bit concerned about the one-fold increase threshold used for ChIP signal. This threshold seems too low to reduce background noise and the analysis may benefit from using a threshold FC>1.2 - 1.5x (i.e. 20-50% increase from background).
RESPONSE: We apologize for the mistake, but we meant Log2FoldChange which is the output of epic2. Thus a log2FC>1 indicates over a 100% increase from background. We corrected this nomenclature over the paper, and including log2FC>1 number of peaks in new Fig. 2D and S2D.

8. Could the authors specify "H3K27me3" throughout manuscript. There are some references to "H3K27" or "H3K27 methylation", which should be replaced.
RESPONSE: We edited the text as suggested.

9. Could the authors please make Fig S2 more presentable. This could probably be combined with Fig S1.
RESPONSE: We have redrawn the Venn diagram and included it in the new Fig. S3 together with new ChIP-seq inflorescences analyses.

10. Fig 4C - If high/med/low expression levels were based on inflorescence tissue, I would have expected more "lowly" expressed genes in the explored quadrant (down-regulated genes with increased H3K27me3). Instead there are many med-highly expressed genes in the "down-regulated" portion of the figure. There are also a number of "no_expr" in the up-regulated gene set. I am unsure how to reconcile this except to ask the authors to reproduce this figure/analysis (e.g. is it possible up- and down-regulated genes were switched or the contrast performed was relative to leaf instead of inflorescence?).
RESPONSE: We think that reviewer #1 misunderstood the performed contrast. In the Fig. 4C, the expression and H3K27me3 changes are relative to leaves. To make it clear we edited figure axes and the text along the results to help the reader.

The p.adj-cutoff used here also seems higher than the commonly, yet arbitrary, used levels of 0.05 or 0.01. I would also appreciate any comments from the authors on this.
RESPONSE: This work was aimed to investigate broad patterns of expression combined with histone marking, and to capture higher diversity we selected differentially expressed genes with padj<0.1, which is still considered statistically significant. However, we see the point of the review and to be consistent throughout the text we edited the number of reported DEG genes to indicate 14,697 DEG p-adj< 0.1 instead of 13,377 DEG p-adj< 0.05.

11. Can the authors please clarify the 1,724 overlap of genes with changed H3K27me3 and mRNA levels. Were the 4,729 deferential H3K27me3 genes overlapped with 13,377 DEGs to give 1,724 genes? Please also perform a Fisher's exact test for some level of statistical confidence.
RESPONSE: The 1,724 genes referred to the genes both marked and differentially expressed (|M/log2FC|>0.5 and padj<0.1; filtering setting on Github). Nonetheless, we found that this sentence is also confusing and does not add any biological information, so we deleted and edited the text accordingly in the new version of the manuscript. We focused on the 729 loci that showed reduced H3K27me3 and increased expression from leaves to inflorescences. This result is enriched 2.07 fold compared to expectations (hypergeometric tests; p-value = 2.9e-164).

Powered by Editorial Manager® and ProduXion Manager® from Aries Systems Corporation

12. P.12 sentence beginning with "All these developmental abnormalities ..." - please include citation for clarity.
RESPONSE: This part of the text was edited for clarification.

13. A browser shot for H3K27me3 and mRNA at the B. rapa AG loci would be nice.
RESPONSE: Thanks for the suggestion, we added a new Fig. 5 with this data.

14. Please specify ChIP qPCR p. 12 sentence beginning with "We performed ChIP experiments...".
RESPONSE: The text was edited for clarification.

15. An ANOVA with post-hoc tukey tests should be performed for Fig 6 B-C.
RESPONSE: We added statistical tests to all the data in the new Fig. 7. We also redraw figure B to put all ChIP-qPCR under the same scale to clarify the difference between H3K27me3 marked loci and Tubulin locus.

16. Were braA.clf-1 seeds ensured to be homozygous?
RESPONSE: We always used homozygous mutant plants genotyped by PCR and sequencing. We edited the methods to clarify this point.

17. Was RNA integrity checked prior to qPCR and 3' RNA sequencing?
RESPONSE: As a standard routine in the laboratory we always check RNA integrity by agarose gel electrophoresis. In addition for genomic experiments RNA, integrity was determined using a TapeStation system. This information has been added to the manuscript.

18. If possible, could the authors re-analyse their raw qPCR fluorescence data using LinReg PCR (Ramakers et al 2003, Ruijter et al 2009).
RESPONSE: We are unsure of the utility of this suggestion. In our experience LinReg PCR software is not totally reliable. For all calculations we determined the threshold cycle (CT) and used the “2-ΔΔCT method”, which is a widely accepted method for qPCR quantification. However, before using any primer we tested the primer amplification efficiency by doing qPCR reactions of serial dilutions of the target amplicon. All our primers showed amplification between 85-115%. We add this information into the manuscript.

19. P. 17, "A first step of trimming was performed with [52] v0.36.5 ." should be "A first step of trimming was performed with Trimmomatic (v0.36.5) [52].
RESPONSE: We edited the text as suggested.

20. Fig S4: could the authors please clarify how many flowers were tested for WT and clf-1?
RESPONSE: We dissected under the microscope the apical flower from the main inflorescence of 20 mutant plants and found 1 flower with homeotic transformations. We never observed this phenotype in the wild-type plant (>100 flowers). We add this information to the new Fig5S legend. We understand that this number may not be statistically significant, thus we edited the text accordingly. In any case, we believe this information is important to the reader as it is remarkably similar to the Arabidopsis clf mutant phenotype.

Reviewer #2

In this MS, the author demonstrates the profile of H3K27me3 in different organ of Brassica rapa, and uncover its role in plant development with mutant analysis. Though the analysis of H3K27me3 has been done in B. rapa before, it focused only on specific region (Genes Genet Syst. 2016, 91:1-10). This study provides a genomic-wide view of this type of histone modification, enable us to get a comprehensive understanding of its function.
There are also some suggestions listed as below:

1.For high-throughput data, three independent biological repeats are required. Although the author demonstrates the reproducibility of the Epigenomic Analysis pipeline, please make it clear whether the replicate tests has been performed to ensure the accuracy of the results.

Powered by Editorial Manager® and ProduXion Manager® from Aries Systems Corporation

	RESPONSE: We agree with reviewer #2 that biological repeats are important in genomic analyses. In fact, we analyzed three biological replicates for all RNA-seq experiments. ChIP-seq signal is not as variable as RNA expression and less replicates are usually performed. Thus, two biological duplicates for the leaf H3K27me3 ChIP-seq were used. However, in the case of the inflorescence H3K27me3 ChIP-seq data we only studied one biological replicate because this is an heterogeneous material (young and old flowers are mixed). We understand that for the purpose of this study one replicate is enough, because we used inflorescence ChIP-seq data to identify remarkable differences between the two studied organs. In addition, following reviewer #2 suggestions, we have made new replicate test between our samples that are included in a new Fig. S7 and S8.<br><br>2.The H3K27me3 modification analysis has been done in both leaves and inflorescences. The Chip-seq data for inflorescences also deserves a figure to show its details.<br>RESPONSE: We thank reviewer #2 suggestions. Inflorescence ChIP analysis has been added added into the new Fig.S2 and S3.<br><br>3.For figure 5, the mutant and the wild type are in different pictures with different bars. If they are in the same picture, it would be much easier for the comparison. At least their bars should be the same.<br>RESPONSE: Following reviewer #2 suggestion we have edited Fig. 5 to make easier the comparison between wild-type and mutant plants. To aid the reader, we add more plant pictures in the new Fig.S5. Please note that all pictures are from plants grown together.
**Additional Information:**	
**Question**	**Response**
Are you submitting this manuscript to a special series or article collection?	No
**Experimental design and statistics**<br><br>Full details of the experimental design and statistical methods used should be given in the Methods section, as detailed in our Minimum Standards Reporting Checklist. Information essential to interpreting the data presented should be made available in the figure legends.<br><br>Have you included all the information requested in your manuscript?	Yes
**Resources**<br><br>A description of all resources used, including antibodies, cell lines, animals and software tools, with enough information to allow them to be uniquely identified, should be included in the Methods section. Authors are strongly encouraged to cite Research Resource Identifiers (RRIDs) for antibodies, model	Yes

organisms and tools, where possible.<br><br>Have you included the information requested as detailed in our Minimum Standards Reporting Checklist?	
**Availability of data and materials**<br><br>All datasets and code on which the conclusions of the paper rely must be either included in your submission or deposited in publicly available repositories (where available and ethically appropriate), referencing such data using a unique identifier in the references and in the "Availability of Data and Materials" section of your manuscript.<br><br>Have you have met the above requirement as detailed in our Minimum Standards Reporting Checklist?	Yes

Powered by Editorial Manager® and ProduXion Manager® from Aries Systems Corporation

Manuscript
Click here to access/download;Manuscript;Paya-Milans et al R1-HL.docx
Click here to view linked References

Genome-wide analysis of the H3K27me3 epigenome and transcriptome in *Brassica rapa*

Miriam Payá-Milans[1,†], Laura Poza-Viejo[1,†], Patxi San Martín-Uriz[2], David Lara-Astiaso[2], Mark D. Wilkinson[1] and Pedro Crevillén[1,*]

[1]Centro de Biotecnología y Genómica de Plantas (CBGP), Universidad Politécnica de Madrid (UPM) - Instituto Nacional de Investigación y Tecnología Agraria y Alimentaria (INIA), Pozuelo de Alarcón (Madrid), Spain

[2]Centro de Investigación Médica Aplicada (CIMA), Universidad de Navarra, Pamplona, Spain

[*]**Correspondence address**: Pedro Crevillén, Centro de Biotecnología y Genómica de Plantas (CBGP), Universidad Politécnica de Madrid (UPM) - Instituto Nacional de Investigación y Tecnología Agraria y Alimentaria (INIA), Pozuelo de Alarcón (Madrid), Spain. E-mail: crevillen.pedro@inia.es http://orcid.org/0000-0003-1276-9792

[†]Equal contributions.

ORCIDs:

Miriam Payá-Milans, 0000-0002-7718-9011;

Laura Poza-Viejo, 0000-0001-6252-3964;

Mark D. Wilkinson, 0000-0001-6960-357X;

Pedro Crevillén, 0000-0003-1276-9792

Abstract

Background

Genome-wide maps of histone modifications have been obtained for several plant species. However, most studies focus on model systems and do not enforce FAIR data management principles. Here we study the H3K27me3 epigenome and associated transcriptome of *Brassica rapa*, an important vegetable cultivated world-wide.

Findings

We performed H3K27me3 chromatin immunoprecipitation followed by high-throughput sequencing and transcriptomic analysis by 3'-end RNA sequencing from *B. rapa* leaves and inflorescences. To analyze these data we developed a reproducible epigenomic analysis pipeline using Galaxy and Jupyter, packaged into Docker images to facilitate transparency and reuse. We found that H3K27me3 covers about a third of all *B. rapa* protein-coding genes and its presence correlates with low transcript levels. The comparative analysis between leaves and inflorescences suggested that the expression of various floral regulatory genes during development depends on H3K27me3. To demonstrate the importance of H3K27me3 for *B. rapa* development, we characterized a mutant line deficient in the H3K27 methyltransferase activity. We found that *braA.clf* mutant plants presented pleiotropic alterations, *e. g.* curly leaves due to increased expression and reduced H3K27me3 levels at AGAMOUS-like loci.

Conclusions

We characterized the epigenetic mark H3K27me3 at genome-wide levels and provide genetic evidence for its relevance in *B. rapa* development. Our work reveals the epigenomic landscape of H3K27me3 in *B. rapa* and provide novel genomics datasets

and bioinformatics analytical resources. We anticipate that this work will lead the way to further epigenomic studies in the complex genome of Brassica crops.

Background

The epigenome comprises alternative chromatin states that can impact gene activity [1]. These include DNA methylation, the incorporation of histone variants and the post-transcriptional modification of histones – like acetylation or methylation on residues in the histone tails which can ultimately modify the interaction with DNA. Epigenetic marks accumulate in response to internal and environmental cues and persist through mitosis during the life span of the organism. A remarkable finding is the extent of the evolutionary conservation in key regulators and mechanisms across the plant and animal kingdoms suggesting that a very ancient mechanism underlies this epigenetic regulation [2].

The trimethylation of histone H3 lysine 27 (H3K27me3) [2–4] is one of the best examples of epigenetic regulation of the gene expression programs. H3K27me3 generally anticorrelates with gene repression and marks the so-called facultative heterochromatin, a fraction of the genome where gene expression is repressed but can be activated in response to developmental or environmental signals [5]. This epigenetic mark is deposited at target genes by specific histone methyltransferases as part of the Polycomb repressive complex 2 (PRC2). The PRC2 complex, which is conserved from animals to plants, comprises a set of core components and several accessory subunits [5,6]. In the model plant *Arabidopsis thaliana* (hereinafter referred to as Arabidopsis) the core PRC2 subunits are well conserved and H3K27me3 is crucial for plant development [3]. During plant growth, different sets of genes are expressed in different organs or tissues and the PRC2 complex is needed to maintain these gene expression patterns [5]. The exact mechanism through which H3K27me3 represses gene expression is not

fully understood [5,6], but H3K27me3 is considered a hallmark of gene repression because it is tightly associated with gene silencing.

In plants, H3K27me3 is crucial for developmental transitions like gametophyte formation, seed germination and floral initiation [3,4]. In Arabidopsis, at least 20% of protein-coding genes are covered by H3K27me3 in a given organ [7,8]; and similar results have been obtained in rice, maize and the model cereal *Brachypodium distachyon*, suggesting a conserved role of this epigenetic mark in plant development [9–11]. The importance of this histone modification is highlighted by recent reports showing that up to 60% of protein-coding genes are silenced by H3K27me3 in several specific plant cell types [12].

The Brassicaceae or Cruciferae family includes Arabidopsis and several important crops. The *Brassica* genus includes a number of condiments and vegetables as well as economically important oilseed crops. Brassica crops have complex genomes that underwent a whole genome triplication with subsequent genome rearrangements and chromosome reduction after the divergence from a common ancestor with Arabidopsis about 15 million years ago [13,14]. Therefore, the mesohexaploid Brassica genomes like *Brassica rapa* (turnip, field mustard; genome AA), *Brassica nigra* (black mustard; genome BB) and *Brassica oleracea* (cabbage; genome CC) are predicted to encode up to three orthologs of each Arabidopsis gene. Within the *Brassica* genus, the diploid *B. rapa* is considered a model for genomic studies because it has a small genome size that makes up half of the genomes of the allotetraploids *Brassica juncea* (indian mustard; AABB) and *Brassica napus* (rapeseed; AACC), which are relevant oilseed crops worldwide. *B. rapa* displays an extreme morphological diversity and includes economically important vegetables and oilseed crops [15,16]. In addition, the *B. rapa* (Chinese cabbage, Chiifu-401) genome has been fully sequenced and annotated and hundreds of accessions have been re-sequenced [14,16,17].

Despite intense epigenetic research in Arabidopsis, genome-wide epigenomic studies in Brassica crops are scarce. We are interested in understanding the epigenetic regulation underlying key agronomic traits in Brassicaceae. To begin answering this question, here we study the genome-wide levels of H3K27me3 in leaves and inflorescences of *B. rapa* R-o-18, an oilseed variety. We found that H3K27me3 is associated with gene silencing and decorates more than 25% of *B. rapa* protein coding genes. Comparative analyses between leaves and inflorescences show that the expression of a number of floral regulators correlates with dynamic changes in H3K27me3. Phenotypic characterization of a mutant in the *B. rapa* homolog of the histone methyltransferase CURLY LEAF (BraA.CLF) revealed the importance of H3K27me3 for proper *B. rapa* development. Mutant *braA.clf* plants showed reduced H3K27me3 and high expression levels of floral identity genes in leaves resulting in a number of pleiotropic developmental alterations. Our work highlights the importance of H3K27me3 deposition in the regulation of developmental transitions in plants and leads the way to further epigenomic studies in Brassica crops.

Data Description

In this work we obtained the genome-wide profile of the epigenetic mark H3K27me3 in *B. rapa*. We performed chromatin immunoprecipitation followed by high-throughput sequencing (ChIP-seq) from leaves and inflorescences. To correlate histone methylation and messenger RNA (mRNA) levels, we extracted RNA from the same harvested material and 3'-end mRNA high-throughput sequencing (3'RNA-seq) was performed. To uncover the relevance of H3K27me3 in *B. rapa* development, we characterized a tilling mutant line deficient in the H3K27 methyltransferase BraA.CLF. ChIP-seq data (225 million paired-end fragments) and 3'RNA-seq data (75 million single-end reads) were archived at NCBI Sequence Read Archive under the accession number PRJNA542357.

Analyses

A Reproducible Epigenomic Analysis pipeline

To enhance compliance with the FAIR principles (findability, accessibility, interoperability, and reusability) for scholarly digital objects [18], we designed a Reproducible Epigenomic Analysis (REA) pipeline for ChIP-seq and RNA-seq using Galaxy [19], an open web-based platform where each analytical step is formally documented and can be shared and reproduced. We generated Galaxy workflows for the analysis of both ChIP-seq data and RNA-seq data. These workflows were executed on a locally administered Galaxy server via a Docker container image [20]. The Docker technology allowed us to bundle all Galaxy components and tools into a distributable package, which is publicly available for download and execution. Analytical steps that could not be integrated within a Galaxy workflow were captured and documented in Jupyter notebooks [21], an open-source interactive computing environment that allows sharing of code, documentation, and results. These notebooks are also available within a Docker image that runs Jupyter. The REA pipeline is available in the GitHub repository https://github.com/wilkinsonlab/epigenomics_pipeline and in the associated Zenodo release (doi:10.5281/zenodo.3298028).

In Fig. 1, a schematic representation of our REA pipeline is shown. We used well-established tools including Bowtie2 [22] for short-read sequence alignment, HTSeq [23] for feature mapping quantification, epic2 [24] for ChIP-seq peak calling, MAnorm [25] for quantitative comparison of ChIP-seq data, and DESeq2 [26] for differential gene expression analysis. A detailed description of these workflows can be found in the Methods section.

Genome-wide identification of H3K27me3 regions in *B. rapa*

To study the epigenetic landscape of H3K27me3 in *B. rapa* we performed ChIP experiments from leaf and inflorescence samples. Immunoprecipitated chromatin and Input (chromatin extracts not subjected to immunoprecipitation) DNA libraries were sequenced by Illumina technology at 125 bp paired-end reads; more than 30 million fragments were obtained for each sample (Table S1) and sequencing data were analyzed using the REA pipeline. Raw paired-end reads were trimmed to remove low quality bases and short reads, with over 99% of ~123 bp-long reads being maintained. Two of the most commonly used aligners for ChIP-seq analysis are BWA [27] and Bowtie2, which carry out fast mapping of DNA sequences using the Burrows-Wheeler transform method. We tested which of these aligners best suited our samples using the latest *B. rapa* v3.0 Chiifu-401 genome as reference [14,17]. Although BWA yielded comparable results, we obtained a small increase on paired-end mapping efficiency using Bowtie2 (Table S2), and this algorithm was therefore used for the remainder of our genomic analyses in *B. rapa*. Mapping with Bowtie2 against the *B. rapa* v3.0 genome [17] yielded an average 82% mapping rate, where 42-61% of the reads mapped to multiple locations (Table S1), likely reflecting the mesopolyploid nature of the *B. rapa* genome or the abundance of repeated DNA elements. After mapping, duplicated reads were removed and ChIP-seq signal distribution over *B. rapa* genome was visualized and inspected using the Integrative Genomics Viewer (IGV) [28].

Using the REA pipeline, we then determined the overall distribution patterns of H3K27me3 in *B. rapa* leaves. A metagene plot of H3K27me3 ChIP-seq signal showed that, as described in other plant species [7,9,10], H3K27me3 is not enriched at promoter regions but rather covers *B. rapa* coding regions, showing little preference for the 5' versus 3' ends of genes (Fig. 2A). A heat map representation of the genome-wide ChIP-seq signal showed that H3K27me3 accumulation displays a gradual variation between genes: with some genes showing high histone methylation density and others relatively low H3K27me3 levels (Fig. 2B). To precisely determine the location of H3K27me3-

marked regions across the genome, we performed a ChIP-seq peak calling analysis adjusting software settings for detection of broad range peaks. We compared two widely-used but different peak-calling algorithms: MACS2 [29], an algorithm initially designed to identify sharp peaks but extended to detect broad peaks such as those arising from this analysis; and epic2, a highly performant implementation of SICER [30], an algorithm designed for noisy and diffuse ChIP-seq data such as histone methylation. Both peak finding algorithms detected a comparable number of peaks (MACS2 20,000 and epic2 15,000 peak regions). However epic2 was able to detect wider histone methylated regions than MACS2 (Fig. S1), with a mean peak size of 2,991 bp for epic2 compared to 1,985 bp for MACS2, and was the preferred tool for studying diffuse epigenetic marks in our study.

Our REA pipeline identified 15,136 H3K27me3 marked regions in *B. rapa* leaves using epic2 (Tables S3 and S4), where 68% overlapped with one or more protein-coding genes (Fig. 2C and D, Table S3). We found 12,480 genes marked with H3K27me3, which represents 27% of all *B. rapa* protein-coding genes. In animals H3K27 methylated regions cover hundreds of kilobases and usually span several genes [6,7]. We found that the average size of *B. rapa* H3K27me3-marked regions was about 3 kb, and that 55% of these peak-regions were associated with single genes (Fig. 2C and D, Table S3). This is similar to what has been described for Arabidopsis [7]. However, consistent with the complexity of the *B. rapa* genome, we also found about 2,000 multigenic regions including some large H3K27 methylated regions spanning more than 10 kb (Table S4). The same overall conclusions were drawn when *B. rapa* inflorescences H3K27me3 ChIP-seq data were analyzed (Fig. S2, Tables S3 and S5). In summary, our analysis indicates that H3K27me3 marks a great portion of the *B. rapa* genome but it is mainly associated with single genes.

H3K27me3 correlates with low transcript levels in *B. rapa*

To identify the correlation between H3K27me3 and gene expression, we performed 3'RNA-seq on the same leaf and inflorescence samples used for ChIP-seq. Profiling RNA levels by 3'RNA-seq and other tag-based methods usually gave higher dynamic detection range and improved mRNA quantification than full transcriptome sequencing [31]. Following the REA pipeline, RNA-seq reads were mapped with Bowtie2 (mapping rate 77% inflorescences and 86% leaves, Table S1), and counted to obtain quantitative mRNA data from *B. rapa* both organs (Table S6). After normalization of expression data, genes were sorted into four categories according to their mRNA levels and used to separately represent their average ChIP-seq H3K27me3 signal on a metagene plot. As shown in Fig. 3A and S3A, genes with high levels of H3K27me3 usually exhibit low or no expression, indicating that, as in most eukaryotes, H3K27me3 in *B. rapa* is associated with gene silencing.

epic2 reports regions that pass a score threshold for ChIP-seq read enrichment over input. To select for genes potentially regulated by H3K27me3 in *B. rapa*, we selected high-confidence peak regions where the ChIP signal determined by epic2 was greater than a two-fold increase over the background (log2FC>1). This analysis identified 8,510 and 7,445 high-confidence H3K27me3 marked genes in leaves and inflorescences respectively that showed notable overlap (Fig. S3B). These gene lists were used as input for Singular Enrichment Analysis (SEA) of Gene Ontology (GO) terms using agriGO [32]. The resulting GO term list was summarized and reduced in complexity using REVIGO [33]. The results in Fig. 3B and S3C show that in both plant organs, in *B. rapa*, H3K27me3-marked genes are enriched in biological process categories related to metabolism (GO:0019222, GO:0044237), biological regulation (GO:0065007), response to stimulus (GO:0050896), transport/localization (GO:0006810, GO:0051179), development (GO:0032502, GO:0040007) and reproduction (GO:0000003). In addition to response to stimulus, H3K27me3-marked genes in leaves were enriched in response to stress (GO:0006950) and response to endogenous stimulus (GO:0009719) in

inflorescences, suggesting that this epigenetic mark plays a role coordinating genomic responses to either external or internal cues. Genes related to metabolism are marked differently, lipid metabolism genes (GO:0006629) were enriched in genes leaves, whereas carbohydrate and secondary metabolism GO categories (GO:0008152, GO:0005975, GO:0019748, GO:0009058) were enriched in inflorescences.

In several eukaryotic organisms, H3K27me3-mediated silencing regulates the transition between developmental programs [2,3]; consistently, genes with development and reproduction functions are significantly marked by both organs. In inflorescences, we found that H3K27me3 marked genes were enriched in GO categories related to cellular/organism processes (GO:0009987, GO:0030154, GO:0051704, GO:0007154). Remarkably, we found that a significant number of genes repressed by H3K27me3 in *B. rapa* leaves were related to transcription (GO:0006351, GO:0010468), cell growth (GO:0016049), and more specifically to flower development (GO:0009908) categories. These data led us to explore how H3K27me3 contributes to the silencing of floral developmental genes in *B. rapa* leaves.

Dynamic changes in H3K27me3 are associated with the floral transition in *B. rapa*

The transition from vegetative to reproductive development is a crucial step in the plant-life cycle [34]. This process is highly regulated and involves a genome-wide transcriptional reprogramming where different developmental programs are activated or repressed [8,12]. To compare H3K27 methylated genes between leaves and inflorescences, we selected the high-confidence peaks from these two organs and performed a quantitative ChIP-seq signal comparison using MAnorm. MAnorm generates a set of representative peaks after merging overlapping peaks between samples and quantifies their read density; values from both organs were compared on an M-A plot and normalized with lowess regression. Setting a |M|>0.5 cut-off (where M

represents the log2 fold-change of the normalized read densities in leaves relative to inflorescences), we found 5,986 differentially H3K27me3-marked regions between leaves and inflorescences including 4,729 differentially marked genes (Fig. 4A and Table S7). Thus, about 55% of all 10,726 merged peaks (from both leaves and inflorescences) showed a significant histone methylation change between these two plant organs.

H3K27me3 changes are not always correlated with gene expression changes [2,6]; *e.g.*, a reduction of H3K27me3 levels may not be enough to promote transcription in the absence of a specific transcription factor. To examine the extent to which the observed H3K27me3 differences in *B. rapa* were correlated with the gene expression changes required for the formation of flowers, differentially expressed genes (DEG) between leaves and inflorescences were determined with our RNA-seq data analysis workflow. Using DESeq2 under our REA pipeline, we found 14,697 DEG (Table S4 and Fig. 4B; |log2FC|>0.5, p-adj<0.1) comparing mRNA levels in leaves relative to inflorescences, indicating that about 32% of *B. rapa* protein-coding genes are differentially expressed between these organs. To study the correlation between differential H3K27me3 deposition and differential mRNA levels between leaves and inflorescences, we represented the differences in H3K27me3 signal (MAnorm M value) against the differential expression levels (DESeq2 log2FC) between leaves and inflorescences (Fig. 4C). This revealed 729 loci with reduced H3K27me3 levels (M>0) and increased mRNA expression in inflorescences (DESeq2 log2FC<0) (Figure 5 and Table S8). Interestingly, SEA analysis of this set of H3K27me3 regulated genes showed that the primary enriched GO terms were related to processes involved in the development of the flower: floral whorl development (GO:0048438), floral organ development (GO:0048437) and flower development (GO:0009908) (Fig 4D). Very interestingly, this list was enriched in well-known *B. rapa* floral regulators like *B. rapa FT*-like genes [35] and more than 60 MADS-box genes. MADS-box proteins are conserved transcription factors that play an important role in the control of flowering time and

reproductive development in plants [35]. The list of H3K27 regulated genes in Table S8 includes a number of *B. rapa* MADS-box genes with homology to floral homeotic genes like *AGAMOUS* (Fig. 5), *APETALA1, CAULIFLOWER, PISTILLATA* and *SEPALLATA* genes, or involved in the floral transition like *AGL19, FRUITFUL* and *SOC1* [36]. All these data suggest that H3K27me3 is important for the expression of floral regulatory genes and, eventually, the proper development of the reproductive structures in *B. rapa*.

H3K27me3 deposition is crucial for *B. rapa* development

In Arabidopsis, there are three H3K27me3 methyltransferases: the main one is CURLY LEAF (CLF) [3,37], which is partially redundant with SWINGER [38], and MEDEA which is only expressed in the female gametophyte and seeds [39]. CLF mutations result in defective floral morphology, early flowering and severe leaf developmental alterations [37]; these developmental defects are due to missexpression of H3K27me3-silenced target genes [38,40].

In the *B. rapa* genome there is one CLF, one SWINGER, and two MEDEA-like homologs [41]. To determine the effect of impaired H3K27 deposition on the development of *B. rapa*, we studied the function of *BraA.CLF* (*BraA04g017190*.3C). Consistent with a possible role as a general histone methyltransferase across the plant, we found that *BraA.CLF* (*BraA04g017190*.3C) gene is expressed in *B. rapa* leaves and flowers (Fig. S4). We obtained a tilling mutant line [42], *braA.clf-1* (Gln615Stop) that produced a truncated protein without the catalytic domain. Mutant *braA.clf-1* plants have a smaller overall size, reduced expansion of flowers, and curled sepals and petals (Fig. 6 and S5). In addition, some mutant flowers showed homeotic transformation of floral organs (Fig. S4C). However, the most striking phenotype of the mutant was the upward curled leaves that were most severe on younger leaves (Fig. 6D and S5A). The small plant size, altered floral development and curled leaves resemble the Arabidopsis *clf* mutant [37], suggesting a high degree of functional conservation between both species.

The upward curved leaves in Arabidopsis *clf* mutant are due to the upregulation of the floral identity gene *AGAMOUS (AG)*, which is a direct CLF target in the leaf [37,43]. In our genomic analysis (Figure 5 and Table S8), we found that the two *B. rapa AG* loci [44], *BraA01g010430.3C* (*BraA.AG.a*) and *BraA03g048590.3C* (*BraA.AG.b*), showed lower expression and higher H3K27me3 in leaves than inflorescences. Thus, we wondered whether BraA.CLF may be repressing these two key floral regulator genes in *B. rapa* leaves. We performed ChIP experiments and tested discrete chromatin regions by real-time quantitative PCR (ChIP-qPCR), and found that H3K27me3 levels were reduced at *BraA.AG.a* and *BraA.AG.b* loci in mutant *braA.clf-1* leaves (Fig. 7A and B). This reduction in H3K27me3 was associated with increased *BraA.AG.a* and *BraA.AG.b* mRNA levels in *braA.clf-1* mutant leaves as determined by real-time quantitative reverse transcription polymerase chain reaction (RT-qPCR) (Fig. 7C). All these data indicate that BraA.CLF is a major histone methyltransferase regulating the deposition of H3K27me3 at key developmental genes in *B. rapa*; and, more importantly, that the correct H3K27me3 is crucial for the optimal growth of Brassica crops.

Discussion

In this work, we determined the genome-wide and organ-specific distribution of H3K27me3 together with the transcriptome dynamics in leaves and inflorescences of *B. rapa* R-o-18, an oilseed cultivar. We found that H3K27me3 is present in a large number of genomic locations across the *B. rapa* genome, mainly associated with protein-coding genes. Interestingly, SEA analyses showed that H3K27me3 marked genes are enriched in GO terms related to gene regulation, such as “regulation of gene expression” (GO:0010468). In parallel with our ChIP-seq experiments, to correlate histone modification levels with transcript levels, we performed mRNA quantification by 3’RNA-seq. As in other organisms, we found that H3K27me3 is anticorrelated with mRNA levels indicating that this epigenetic mark is also a hallmark for gene inactivation in *B. rapa.* All

these data suggest that H3K27me3 plays an important role in the regulation of gene expression in *Brassica* crops.

Our analysis also revealed that a number of genes related to floral development were marked by H3K27me3 in *B. rapa* leaves. Floral development and the proper formation of the reproductive organs of the plant are crucial for the formation of fruits and seeds, and have a great impact on crop yield. To find out the role of H3K27me3 on floral development in *B. rapa*, we performed a differential analysis of ChIP-seq and 3'RNA-seq signal in leaves vs inflorescences. We found that genes involved in the floral transition or floral organ identity are highly methylated and not expressed in leaves; whereas in inflorescences, the high mRNA levels of this group of genes was associated with a significant decrease of H3K27me3. These data indicate that the repressive H3K27me3 mark is playing a role to prevent ectopic expression of floral regulator genes outside reproductive tissues.

The trimethylation of histone H3K27 is performed by conserved Polycomb PRC2 complexes. In the model plant Arabidopsis, CLF is the main H3K27 methyltransferase. In this work, we isolated a tilling mutant line on the sole *B. rapa* CLF homolog. Phenotypic analysis showed that *braA.clf-1* mutant plants display several developmental defects like a characteristic curved leaf phenotype. Further ChIP and RT-qPCR experiments showed that *BraA.AG.a* and *BraA.AG.b*, the two *B. rapa* homologs of the floral identity gene *AG*, were upregulated due to reduced H3K27me3 in *braA.clf-1* mutant leaves. All these data suggest that BraA.CLF is a *bona fide* H3K27 methyltransferase and demonstrate the important role of this epigenetic mark for the correct development of *B. rapa* plants.

The *Brassica* genus contains a diverse collection of economically important crop species, including the third-highest produced oil crop in the world and constituting an increasingly popular source of nutrients due to their anticancer, antioxidant, anti-inflammatory properties and high nutritional value [15]. The *B. rapa* genome was the first sequenced Brassica crop [14], but in recent years the genome sequences of cabbage

(*B. olearacea*), black mustard (*B. juncea*) and rapeseed (*B. napus*) have been released [45]. The comprehensive study of H3K27me3 in *B. rapa* presented here, opens the door to explore other Brassica crops with more complex genomes. We hope that the epigenomic datasets and bioinformatic analytical pipelines generated in this work will aid future studies to shed more light on the epigenetic regulation in plants.

Potential implications

Here we presented novel H3K27me3 epigenomic and transcriptomic *B. rapa* datasets. To understand the molecular mechanisms of epigenetic regulation in plants is important as fundamental knowledge but also has enormous implications for crop improvement and agriculture [46]. DNA sequence variability alone cannot explain all the diversity of plant phenotypes [47]. The isolation of stable epigenetic variants of several crops has led to the idea that epigenetics could contribute to heritable natural variation that can be selected in plant breeding and crop improvement programs [46,47].

To facilitate transparency and reproducibility, the bioinformatic pipeline we generated for ChIP-seq and RNA-seq - the Reproducible Epigenomic pipeline - has been made publicly available, together with the output data being extensively annotated in conformance with the FAIR data principles. The pipeline was constructed using a set of well-established genomic tools and approaches, using a combination of a Galaxy environment and Jupyter notebooks [21]. Both Galaxy and Jupyter provide analytics environments that can be reproduced by anyone through a user-friendly Web interface, providing a platform for others to execute similar analyses following our approach.

Methods

Plant Material

B. rapa yellow sarson (*ssp. trilocularis*) R-o-18 wild-type and *braA.clf-1* (ji32391-a; Gln615Stop) mutant seeds were obtained from RevGenUK [42]. For *braA.clf-1* characterization, we used sibling homozygous mutant plants derived from a backcross to the parental line; all *braA.clf-1* plants were genotyped by PCR and sequencing using the primers in Table S9. Plants were sown in 15 cm diameter plastic pots containing a mixture of substrate and vermiculite (3:1) with added controlled-release fertilizer (*Nutricote,* Projar Ltd.); and then grown in controlled-environment plant growth chambers with day/night temperatures of 21/19°C, a mix of cool-white and wide-spectrum FLUORA fluorescent lights (100 µE/m^{2}s) and 16 h of light followed by 8 h of darkness. Pictures of flowers were taken using a Leica MZ10 F Stereomicroscope.

Chromatin Immunoprecipitation and DNA sequencing

ChIP experiments were performed using *B. rapa* R-o-18 wild-type primary leaves collected 14 days after germination (DAG) (Fig. S6A), and inflorescences collected when the first flowers started to open (Fig. S6B and C). Sampling was performed at the end of the light period (zeitgeber time ZT16). Each biological replicate comprises samples from 6 independent plants. ChIP experiments were performed using 2 g of tissue as described at our Plant Chromatin Immunoprecipitation protocol V.2 [48]. We used a specific antibody against H3K27me3 (Millipore 07-449, Lot No. 2736613.). The primers used for the ChIP-qPCR amplification reactions can be found in Table S9.

ChIP DNA was quantified by Qubit 3.0 Fluorometer (Life Technologies) and ChIP-seq sequencing libraries were prepared using NEBNext Ultra DNA Library Prep kit (New England BioLabs 7370) starting from 4 ng of immunoprecipitated DNA. Libraries quality was determined with a Bioanalyzer instrument (Agilent) and concentration was determined using the KAPA Library Quantification Kit (ref. KK4835, KapaBiosystems). One inflorescence and two leaves ChIP-seq biological replicates were sequenced at

2x125 bp paired-end reads in an Illumina HiSeq 2500 platform using the SBS v4 kit (Illumina) at the Genomics Units of CNAG-CRG (Barcelona, Spain).

Gene expression profiling and 3'RNA sequencing

Total RNA was extracted from non-crosslinked aliquots of the same harvested plant tissues used for ChIP. We used EZNA Plant RNA Kit (Omega Bio-tek) following the manufacturer's recommendations. Each biological replicate contained samples from 6 independent plants. RNA integrity was checked by agarose gel electrophoresis and complementary DNA (cDNA) was prepared by reverse transcription of 1 µg of total RNA using "Maxima cDNA Kit with dsDNase" (Thermo Fisher Scientific) according to the manufacturer's instructions, and quantification was performed by RT-qPCR using the LightCycler 480 and SYBR Green I Master mix (Roche). Gene expression was determined by $2^{-\Delta\Delta CT}$ method using *BraA.TUBULIN* (*BraA10g026070.3C*) [49] as housekeeping gene. The primers used for RT-qPCR primers can be found in Table S9.

Libraries for 3'RNA-seq were prepared using a MARS-seq protocol adapted for bulk RNA-seq [50,51] with minor modifications. Briefly, RNA quality was determined using a TapeStation 4200 system (Agilent) and 100 ng of total RNA were reverse-transcribed using poly-dT oligos carrying a 7 bp-index. cDNAs were then pooled and subjected to linear amplification via *in vitro* transcription. The resulting amplified RNA was fragmented and dephosphorylated. Ligation of partial Illumina adaptor sequences [50] was followed by a second reverse-transcription and full Illumina adaptor sequences were added during a final library amplification. Libraries were quantified using a Qubit 3.0 Fluorometer (Life Technologies) and their size profiles examined in a TapeStation 4200 system (Agilent). Three biological replicates for each organ were sequenced at 1x68 bp single-end reads in an Illumina NextSeq 500 (Illumina) at the Advanced Genomics Laboratory of the Centro de Investigación Médica Aplicada (CIMA; Pamplona, Spain).

The Reproducible Epigenomic Analysis pipeline

Analysis of sequencing data was conducted within environments that either allow the assembly of bioinformatics tools into analytical workflows (Galaxy), or serve to share interactive code-containing documents (Jupyter Notebooks). Jupyter Lab's R and bash kernels were installed using Anaconda3 (Anaconda Software Distribution, 2018). The REA pipeline was implemented as a series of steps distributed within a Docker container (https://hub.docker.com/) which includes all required software dependencies. Any user can deploy a REA instance on-demand. To be able to download and use a dockerized version of Galaxy (https://github.com/bgruening/docker-galaxy-stable), Docker version 18.09.3 was first installed following the documentation on Docker-CE for Ubuntu. Next, Galaxy version 18.05 was locally installed with the commands:

```
mkdir -p ~/DockerFolders/galaxy_v1
docker run -d -p 8080:80 \
  -v ~/DockerFolders/galaxy_v1/:/export/ \
  quay.io/bgruening/galaxy:18.05
```

This local Galaxy server can be accessed and administered on http://localhost:8080/. Most tools were installed from the Galaxy Tool Shed, with the exception of epic2 v0.0.14 (https://github.com/biocore-ntnu/epic2), which we manually installed inside the docker container via an interactive session using python pip and wrapped as a Galaxy tool. Our wrapped epic2 tool has been successfully integrated into Galaxy and published into the Galaxy Tool Shed (www.galaxygroject.org). Versions and settings of tools used in Galaxy are noted in the workflows published in our DockerHub images, as well as described below.

To facilitate reproducibility of our data analyses, we built and published two Docker images, which contained all dependencies and software requirements to create

a ready-to-run analytical workflow (see Availability of Supporting Data). The first image contains a Galaxy instance with required tools installed and accessory files to download/index the *B. rapa* reference genome and prepare/run workflows with the analytical steps described below. These workflows are designed to download raw sequencing reads from SRA and export the results locally for further processing. The second image, which runs Jupyter Lab, includes software installations and notebooks with instructions to finalize data analysis and explore results; they include, as a default view, the results published here. These images are designed to operate on a shared local directory for Jupyter to access Galaxy results. Detailed instructions on how to deploy and run each container can be found in our GitHub repository README file.

To enhance our compliance with FAIR publishing requirements, metadata files following schema.org format for both the ChIP-Seq and RNA-Seq experiments are available in the GitHub. In addition, metadata descriptors for the ChIP-seq and RNA-seq data submissions in the NCBI SRA are available in ISA-Tab format in the same GitHub repository. All of these files are also available in the associated Zenodo snapshot release (doi:10.5281/zenodo.3298028).

ChIP-seq analysis

Sequencing data was uploaded to a local Galaxy instance in fastqsanger format. Each sample was independently processed and replicates pooled for peak calling. A first step of trimming was performed with (v0.36.5) [52]. Trimmed reads were mapped to the *B. rapa Chiifu* v3.0 genome using Bowtie2 v2.3.4.2 (Bowtie, RRID:SCR_005476) or BWA v0.7.17.3 (BWA, RRID:SCR_010910), and the results were compared with SAMtools Flagstat [53]. Bowtie2 aligned reads were used in subsequent analyses. BAM files were filtered with SAMtools v1.8 (SAMTOOLS, RRID:SCR_002105) by mapping quality (including concordance of mates) and by duplication state (possible duplicate reads that may arise during library preparation), marked by Picard MarkDuplicates v2.18.2.0 [54].

The set of deduplicated reads was used for ChIP-seq peak calling on pooled replicates using epic2 v0.0.14 or MACS2 v2.1.1 for comparison to one another; the epic2 output was then used for downstream processes. Additional steps in the workflow are aimed at collecting quality metrics MultiQC [55], as well as producing bigwig files using DeepTools 3.1.2 (Deeptools, RRID:SCR_016366) [56] with the coverage of filtered alignments on bin sizes of 50 bp.

Results from data analysis on Galaxy were downloaded locally for further processing and visualization. The *B. rapa* genome v3.0 contains 10 chromosomes but also thousands of smaller scaffolds, with sequences from either nuclear chromosomes or chloroplast/mitochondria organelles. Thus, prior to analysis of differential H3K27me3 levels between leaves and inflorescences, raw peak calling results from epic2 were curated via selection of nuclear regions and visual inspection on IGV viewer. Differential levels of H3K27me3 histone mark intensities were computed by comparison of read abundances on our curated list of peaks with MAnorm v1.2.0 (MAnorm, RRID:SCR_010869) [25], which uses MA plot methods to normalize read density levels on provided peaks and calculate *p*-values. This comparison is performed using all available reads, including duplicates, resulting in a list of common peaks with the differences on leaf compared to inflorescences. Peaks from either epic2 or MAnorm were annotated according to overlap of *B. rapa* gene models using ChIPpeakAnno (ChIPpeakAnno, RRID:SCR_012828) [57], and the distribution of ChIP signal over genes was visualized with ngs.plot (ngs.plot, RRID:SCR_011795) [58]. To test replicate samples, read density was obtained over bins of 10 kb of the *B. rapa* genome and normalized using scaling factors. These counts were plotted and sample-sample correlations were calculated using Spearman method, on scatter plots with density curves (plot instructions as described in [59]) (Fig. S7).

RNA-seq analysis

The bulk transcriptomic analysis was performed using a specific RNA-seq analysis workflow on a local Galaxy instance. Single-end reads were trimmed with Trimmomatic and mapped with Bowtie2 against *B. rapa* v3.0 genome. Before counting, available *B. rapa* v3.0 gene models, which only comprise the coding sequences, were extended by 300 bp at each gene's 3' UTR regions and used to quantify aligned reads using HTSeq-count script with stranded and intersection of non-empty sets options. The obtained counts were used for mRNA differential expression analysis with DESeq2 1.18.1 (DESeq2, RRID:SCR_015687) to infer gene expression changes of leaves compared to inflorescences. DEseq2 results were downloaded locally for further analysis outside Galaxy in combination with annotated peaks. To test replicate samples, normalized counts were used to visualize sample-sample correlation and calculate the Spearman correlation (Fig. S8) [59]. Categorization of genes by expression level was achieved after transformation of count data into z-scores as follows: for each expressed gene and organ, normalized counts were averaged across replicates, subtracted the dataset average and divided by dataset standard deviation. These z-scores were used to define genes with low ($z < -0.5$), medium ($-0.5 < z < 0.5$), and high expression ($z > 0.5$).

Other Bioinformatic analyses

Gene Ontology analysis was performed using agriGO v2.0 (agriGO, RRID:SCR_006989) [32] (Fisher statistical test method; Yekutieli Multi_test adjustment method; $p < 0.05$; and Plant GO slim ontology type); data was visualized reduced in complexity and redundant GO terms using REVIGO (REViGO, RRID:SCR_005825) [33] with default parameters (allowed similarity = 0.7; semantic similarity measure = SimRel).

Functional annotation of *B. rapa* v3.0 gene models was obtained from [17]. Custom annotation of gene models from *B. rapa* genome v3.0 was obtained blasting coding sequences against *B. rapa* genome v1.5 (E-value cutoff of 0.001) and

Arabidopsis (TAIR10 proteins, blastx of *B. rapa* coding sequences with an E-value cutoff of 1e-25).

During our analyses of H3K27me3 levels on *B. rapa* AGAMOUS genes, we found that *BraA.AG.a* (*BraA01g010430.3C*) gene structure annotation was not correct in the recent *B. rapa* genome v3.0. We curated *BraA.AG.a* gene structure using AUGUSTUS (Augustus, RRID:SCR_008417) [60] and Bra013364 (*B. rapa* genome V1.5) gene information at *B. rapa* database [45].

Availability of supporting source code and requirements

- Project name: Epigenomics Workflow on Galaxy and Jupyter
- Project home page: https://github.com/wilkinsonlab/epigenomics_pipeline
- Operating systems: Platform independent
- Programming languages: Python, R, Bash
- Other requirements: none
- License: MIT
- RRID: SCR_017544

Availability of supporting data

ChIP-seq and RNA-seq data sets supporting the results of this article are available at NCBI Sequence Read Archive under the accession number PRJNA542357. Latest versions of the components of the REA pipeline, and instructions to deploy the Galaxy/Jupyter containers and run the analysis can be found in the GitHub repository https://github.com/wilkinsonlab/epigenomics_pipeline; this is associated with a Zenodo release to match the configuration used in this publication [61]. The REA pipeline is registered as RRID:SCR_017544 and biotools:Epigenomics_Workflow_on_Galaxy_and_Jupyter at SciCrunch and bio.tools

databases, respectively. Compiled docker images are available at https://hub.docker.com/u/mpaya. All supporting data are available in the *GigaScience* GigaDB database [62].

Additional files

Additional file 1 Figure S1: Differences between epic2 and MACS2 peak calling algorithms.

Additional file 1 Figure S2: ChIP-seq analysis of H3K27me3 regions in B. rapa inflorescences.

Additional file 1 Figure S3: Comparisons of H3K27me3 ChIP-seq signal in *B. rapa* inflorescences

Additional file 1 Figure S4: Transcript levels of *B. rapa* H3K27 methyltransferase encoding-genes.

Additional file 1 Figure S5: *BraA.clf-1* mutant phenotypes.

Additional file 1 Figure S6: Pictures of *B. rapa* plant sampling materials.

Additional file 1 Figure S7. ChIP-seq sample-sample correlation tests.

Additional file 1 Figure S8. RNA-seq sample-sample correlation tests.

Additional file 2 Table S1. Alignment statistics.

Additional file 2 Table S2. Comparison of Bowtie2 and BWA performance.

Additional file 2 Table S3. H3K27 trimethylated regions identified in *B. rapa*.

Additional file 3 Table S4. List of H3K27me3 peaks in *B. rapa* leaves.

Additional file 3 Table S5. List of H3K27me3 peaks in *B. rapa* inflorescences.

Additional file 3 Table S6. 3'RNA-seq results.

Additional file 3 Table S7. List of H3K27me3 differentially marked regions

Additional file 3 Table S8. H3K27me3 regulated genes.

Additional file 4 Table S9. Primer list.

Declarations

The authors declare that they have no competing interests.

Abbreviations

3'RNA-seq: 3'-end mRNA high-throughput sequencing

AG: AGAMOUS

cDNA: complementary DNA

ChIP: chromatin immunoprecipitation

ChIP-seq: ChIP followed by high-throughput sequencing

ChIP-qPCR: ChIP followed by real-time quantitative polymerase chain reaction

CLF: CURLY LEAF

DAG: days after germination

DEG: differentially expressed genes

FAIR: findability, accessibility, interoperability, and reusability

H3K27me3: histone H3 lysine 27 trimethylation

IGV: integrative genomics viewer

Kb: Kilobase

log2FC: the base *2* logarithm of the fold-change

M: log2 fold-change of the normalized H3K27me3 read densities in leaves relative to inflorescences calculated by MAnorm.

mRNA: messenger RNA

PRC2: Polycomb repressive complex 2

REA: reproducible epigenomic analysis

RT-qPCR: real-time quantitative reverse transcription polymerase chain reaction

SEA: singular enrichment analysis

s.d: standard deviation

Competing interests

The authors declare that they have no competing interests.

Funding

This work was supported by grant BIO2015-68031-R and grant RYC-2013-14689 to PC, and BES-2016-078939 fellowship to LP from the Spanish Ministerio de Economia y Competitividad (MINECO/FEDER, EU); and by the "Severo Ochoa Program for Centres of Excellence in R&D" from the Agencia Estatal de Investigación of Spain (grant SEV-

2016-0672 (2017-2021) to the CBGP. MPM was supported by a Postdoctoral contract associated to the Severo Ochoa Program.

Author Contributions

PC conceived the work; MPM performed computational biology analyses; LPV performed initial bioinformatic analysis and experimental research. PSMU and DLA performed 3'RNA-seq library preparation and sequencing. MW contributed with analytical tools and metadata generation. PC and MPM wrote the first draft of the manuscript that was completed with the assistance of LPV and MW. All authors approved the final version of the article.

Acknowledgements

We are grateful to Jose A. Jarillo (CBGP, Madrid), Manuel Piñeiro (CBGP, Madrid) and Martín Trick (John Innes Centre, UK) for their help along this project. We thank Laura Castro (CIMA, Spain) for RNA-seq data processing. The authors thank the CNAG-CRG (Barcelona, Spain) for assistance with ChIP-seq experiments. We thank Xiaowu Wag (Institute of Vegetables and Flowers, China) for *B. rapa* gene ontology annotations and Abdul Baten and Graham King for sharing information about *B. rapa* R-o-18 genome.

FIGURE LEGENDS

Figure 1: Schematic view of the analytical workflow of the Reproducible Epigenomic Analysis pipeline (REA). A) Samples from leaves and inflorescences were used for ChIP-seq and RNA-seq. B) Major analytical steps were conducted in a reproducible Galaxy workflow, running on a Docker container. C) Further analysis and graphical representation of results were tracked and run on Jupyter interactive notebooks.

Figure 2: ChIP-seq analysis of H3K27me3 regions in *B. rapa* leaves. A) Metagene plot with the average profile of mean ChIP-seq signal over marked genes. B) Gene heat map of H3K27me3 marked genes, scaling genes to a same size flanked by 1 kb regions. Color scale indicates the log2 ratios of ChIP samples compared to input. C and D) Distribution of H3K27me3 peak regions by length (C) and number of covered genes (D). Total and high-confidence (log2FC>1) H3K27me3 peaks are represented in (D).

Figure 3: Influence of H3K27me3 on gene function. A) Metagene plot of H3K27me3 ChIP-seq signal in genes categorized by gene expression level in *B. rapa* leaves. Genes were classified as high, medium, low and no expression based on their normalized RNA-seq levels. B) Semantic clustering of enriched GO terms of H3K27me3 marked genes in *B. rapa* leaves. SEA was performed with agriGO (default background=TAIR10_2017 genome locus) and the significant GO terms were clustered with REVIGO tool. Biological process terms from GO are positioned in a two dimensional space derived by multidimensional scaling to a matrix of the semantic similarity of GO terms. The size of the bubble indicates the frequency of the GO term in the underlying Arabidopsis TAIR10 gene ontology and *p-values* are indicated by a color scale.

Figure 4: Differential accumulation of H3K27me3 between *B. rapa* leaves and inflorescences. A) MA plot showing the differential accumulation of H3K27me3 on ChIP-seq peaks determined with MAnorm; significance is indicated with color scale - red indicates -log10(*p*-value)>50. Normalized read densities of leaves relative to inflorescences were compared to represent the average signal strength of samples (A value) against their log2 fold change (M value) of each peak. B) MA plot showing the comparison of normalized gene expression between leaves and inflorescences

determined with DESeq2; significant DEGs (*p*-adj<0.1) are colored red. C) Distribution of genes marked by H3K27me3 in leaves comparing the differential levels of H3K27me3 and mRNA (*p*-adj<0.1) on leaves versus inflorescences. Dots were colored accordingly to their mRNA level category (high, medium, low and no expression) in inflorescences. D) Top enriched GO terms in the subset of H3K27me3 regulated genes with decreased mark deposition (M>0) and increased gene expression (FC<0) in inflorescences compared to leaves. Enrichment was determined by SEA on agriGO using all DEGs as custom background.

Figure 5: Example of H3K27me3-marked floral regulator loci. IGV viewer snapshots showing the H3K27me3 ChIP-seq and 3'RNA-seq data of *BraA.AG.a* (A) and *BraA.AG.b* (B) in *B. rapa* leaves and inflorescences.

Figure 6: Characterization of *braA.clf-1* mutant. A-B) Pictures of *B. rapa* R-o-18 wild-type (A) and *braA.clf-1* (B) at flowering stage (bar scale 5 cm). C-D) Pictures of *B. rapa* R-o-18 wild-type (C) and *braA.clf-1* (D) at 25 DAG (bar scale 5 cm). E-F) Pictures of *B. rapa* R-o-18 wild-type (E) and *braA.clf-1* (F) flowers (bar scale 1 mm). The *braA.clf-1* mutant shows smaller and curly leaves, and not well expanded flowers with curled sepals and petals.

Figure 7: BraA.CLF represses *BraA.AG.a* and *BraA.AG.b* expression in leaves. A) Cartoon depicting a representation of *BraA.AG.a* and *BraA.AG.b* loci and the chromatin regions analyzed by ChIP-qPCR. B) ChIP-qPCR showing the H3K27me3 levels at *AG*-like genes in *B. rapa* wild-type vs *braA.clf-1* mutant. Histone modification levels as determined by quantitative qPCR amplification, data represent the average of two biological replicates; error bars indicate ±*s.d*. (n=2); * p<0.05 , ** p<0.01, *** p<0.001

(Student´s t-test). ChIP enrichments were quantified as %INPUT normalized to total DNA content determined by QUBIT assay. As negative control H3K27me3 levels at active housekeeping *BraA.TUBULIN* gene (*BraA10g026070.3C*) are shown in the figure. C) RT-qPCR data expression levels of *AG*-like genes in *B. rapa* wild-type vs *braA.clf-1* mutant. Data represent the average of three biological RNA replicates; error bars indicate ±*s.d*. (n=2); *** p<0.001 (Student´s t-test, leaves data comparison).

Additional files

Additional file 1 Figure S1: Differences between epic2 and MACS2 peak calling algorithms. ChIP-seq signal of two representative *B. rapa* chromosomal regions are displayed using IGV viewer. As shown in the figure, both peak calling algorithms resulted in similar results but in some cases MACS2 calls multiple peaks at regions whereas epic2 calls broad peak regions.

Additional file 1 Figure S2: ChIP-seq analysis of H3K27me3 regions in *B. rapa* inflorescences. A) Metagene plot of average ChIP-seq signal over marked genes. B) Gene heat map of H3K27me3 marked genes. Color scale indicates the log2 ratios of ChIP samples compared to input. C and D) Distribution of H3K27me3 peak regions by length (C) and number of covered genes (D). Total and high-confidence (log2FC>1) H3K27me3 peaks are represented in (D).

Additional file 1 Figure S3: Comparisons of H3K27me3 ChIP-seq signal in *B. rapa* inflorescences. A) Metagene plot of H3K27me3 ChIP-seq signal in genes categorized by gene expression level in *B. rapa* inflorescences. B) Venn diagram showing the overlap of genes marked by high-confident H3K27me3 peaks determined by epic2 (log2FC>1) between leaves and inflorescences. C) Semantic clustering of enriched GO terms of

H3K27me3 marked genes in *B. rapa* inflorescences. SEA analysis performed with agriGO (default background=TAIR10_2017 genome locus) and scattered plot generated with REVIGO tool. Biological process terms from GO are positioned in a two dimensional space derived by multidimensional scaling to a matrix of the semantic similarity of GO terms. The size of the bubble indicates the frequency of the GO term in the underlying Arabidopsis TAIR10 gene ontology and *p-values* are indicated by a color scale.

Additional file 1 Figure S4: Transcript levels of *B. rapa* H3K27 methyltransferase encoding-genes. Graphical representation of the data (normalized counts) from our 3'RNA-seq experiments from leaves and inflorescences.

Additional file 1 Figure S5: *BraA.clf-1* mutant phenotypes. A) Pictures of *B. rapa* R-o-18 wild-type (top) and *braA.clf-1* (bottom) plants 12, 15 and 18 DAG. B) Examples of mature *B. rapa* R-o-18 wild-type (left) and *braA.clf-1* (right*)* plants at flowering stage C) Homeotic changes during flower development in *braA.clf-1* plants. The arrow shows a sepal and petal mixture that was found in the main inflorescence of 1 out of 20 mutant plants analyzed. We never observed this organ fusion in wild-type flowers.

Additional file 1 Figure S6: Pictures of *B. rapa* plant sampling materials. Plants 14 DAG (A) and inflorescences at collection time (B).

Additional file 1 Figure S7. ChIP-seq sample-sample correlation tests.

Scatter plots showing sample-sample comparisons of ChIP-seq read counts on genomic bins from inflorescence and leaf ChIP and input samples. We found a 0.94 correlation between duplicated ChIP samples (leaf_c1 vs leaf_c2). The upper-right triangle indicates the Spearman correlation between samples. The middle density diagrams represent per

sample frequency distribution of normalized read counts. The bottom-left triangle displays sample-wise scatter plots of normalized counts per genomic bin with regression line and value.

Additional file 1 Figure S8. RNA-seq sample-sample correlation tests.

Scatter plots showing sample-sample comparisons of normalized RNA-seq read counts on genes from inflorescence and leaf RNA-seq triplicates. We found a 0.96 mean correlation among RNA-seq triplicates. The upper-right triangle indicates the Spearman correlation between samples. The middle density diagrams represent per sample frequency distribution of normalized read counts. The bottom-left triangle displays sample-wise scatter plots of normalized counts per gene with regression line and value.

Additional file 2 Table S1. Alignment statistics. Summary table indicating, for each ChIP-seq and RNA-seq sample, the number of total reads sequenced before and after quality trimming, and different mapping statistics using Bowtie2.

Additional file 2 Table S2. Comparison of Bowtie2 and BWA performance. Mapping statistics of Bowtie2 and BWA alignment of leaves samples.

Additional file 2 Table S3. H3K27 trimethylated regions identified in *B. rapa*. A) General statistics of H3K27me3 peaks. B) Annotation of peaks that overlap with gene models.

Additional file 3 Table S4. List of H3K27me3 peaks in *B. rapa* leaves. InsideFeature column: *overlapStart*, peak on 5' region of gene; *overlapEnd*, peak on 3' end of gene; *inside*, peak located within gene; *includeFeature*, gene is located within peak.

Additional file 3 Table S5. List of H3K27me3 peaks in *B. rapa* inflorescences. InsideFeature column: *overlapStart*, peak on 5' region of gene; *overlapEnd*, peak on 3' end of gene; *inside*, peak located within gene; *includeFeature*, gene is located within peak.

Additional file 3 Table S6. 3'RNA-seq results. Differential expression was calculated on leaves vs inflorescences using DESeq2. Mean counts and standard deviation correspond to three biological replicates; expression level categories were determined from z-score transformations (see Methods); log2FC, padj and direction of change indicate differential expression results.

Additional file 3 Table S7. List of H3K27me3 differentially marked regions. Differentially marked peaks were identified with MAnorm. *M_value*, log2FC of normalized read densities; *A_value*, average signal strength; *Peak_Group*, origin of peaks (one organ or common).

Additional file 3 Table S8. H3K27me3 regulated genes. List of genes that show reduced K3K27me3 (MAnorm M>0.5) and higher expression (log2FC<0.5) in inflorescences than leaves.

Additional file 3 Table S9. Primer list. Sequence of the primers used in this work. Prior all experiments, primers amplification efficiency was determined by qPCR amplification of serial dilutions of DNA target amplicon. All primers showed an amplification efficiency between 85-115%.

References

1. Murrell A, Rakyan VK, Beck S. From genome to epigenome. *Hum Mol Genet*. 2005;14:3–10.

2. Sawarkar R, Paro R. Interpretation of developmental signaling at chromatin: the Polycomb perspective. *Dev Cell*. 2010;19:651–61.

3. Xiao J, Wagner D. Polycomb repression in the regulation of growth and development in Arabidopsis. *Curr Opin Plant Biol*. 2014;23:15–24.

4. Schatlowski N, Creasey K, Goodrich J, Schubert D. Keeping plants in shape: polycomb-group genes and histone methylation. *Semin Cell Dev Biol*. 2008;19:547–53.

5. Margueron R, Reinberg D. The Polycomb complex PRC2 and its mark in life. *Nature*. 2011;469:343–9.

6. Grossniklaus U, Paro R. Transcriptional Silencing by Polycomb-Group Proteins. *Cold Spring Harb Perspect Biol*. 2014;6:a019331–a019331.

7. Zhang X, Clarenz O, Cokus S, Bernatavichute Y V., Pellegrini M, Goodrich J, et al. Whole-genome analysis of histone H3 lysine 27 trimethylation in Arabidopsis. *PLoS Biol*. 2007;5:e129.

8. Lafos M, Kroll P, Hohenstatt ML, Thorpe FL, Clarenz O, Schubert D. Dynamic regulation of H3K27 trimethylation during Arabidopsis differentiation. *PLoS Genet*. 2011;7:e1002040.

9. He G, Zhu X, Elling A a, Chen L, Wang X, Guo L, et al. Global epigenetic and transcriptional trends among two rice subspecies and their reciprocal hybrids. *Plant*

Cell. 2010;22:17–33.
10. Makarevitch I, Eichten SR, Briskine R, Waters AJ, Danilevskaya ON, Meeley RB, et al. Genomic distribution of maize facultative heterochromatin marked by trimethylation of H3K27. *Plant Cell*. 2013;25:780–93.
11. Huan Q, Mao Z, Chong K, Zhang J. Global analysis of H3K4me3/H3K27me3 in Brachypodium distachyon reveals VRN3 as critical epigenetic regulation point in vernalization and provides insights into epigenetic memory. *New Phytol*. 2018;1.
12. You Y, Sawikowska A, Neumann M, Posé D, Capovilla G, Langenecker T, et al. Temporal dynamics of gene expression and histone marks at the Arabidopsis shoot meristem during flowering. *Nat Commun*. 2017;8:15120.
13. Lysak MA, Koch MA, Pecinka A, Schubert I. Chromosome triplication found across the tribe Brassiceae. *Genome Res*. 2005;15:516–25.
14. Wang X, Wang H, Wang J, Sun R, Wu J, Liu S, et al. The genome of the mesopolyploid crop species Brassica rapa. *Nat Genet*. 2011;43:1035–9.
15. Sadowski J. Genetics, Genomics and Breeding of Vegetable Brassicas. Sadowski J, Kole C, editors. *CRC Press*; 2011.
16. Cheng F, Sun R, Hou X, Zheng H, Zhang F, Zhang Y, et al. Subgenome parallel selection is associated with morphotype diversification and convergent crop domestication in Brassica rapa and Brassica oleracea. *Nat Genet*. 2016;1–10.
17. Zhang L, Cai X, Wu J, Liu M, Grob S, Cheng F, et al. Improved Brassica rapa reference genome by single-molecule sequencing and chromosome conformation capture technologies. *Hortic Res*. 2018;5:50.
18. Wilkinson MD, Dumontier M, Aalbersberg IjJ, Appleton G, Axton M, Baak A, et al. The FAIR Guiding Principles for scientific data management and stewardship. *Sci Data*. 2016;3:160018.
19. Afgan E, Baker D, Batut B, van den Beek M, Bouvier D, Čech M, et al. The Galaxy platform for accessible, reproducible and collaborative biomedical analyses: 2018 update. *Nucleic Acids Res*. 2018;46:W537–44.

20. Maerkel D. Docker: Lightweight Linux Containers for Consistent Development and Deployment. *LINUX J*. 2014.

21. Grüning BA, Rasche E, Rebolledo-Jaramillo B, Eberhard C, Houwaart T, Chilton J, et al. Jupyter and Galaxy: Easing entry barriers into complex data analyses for biomedical researchers. Ouellette F, editor. *PLOS Comput Biol*. 2017;13:e1005425.

22. Langmead B, Salzberg SL. Fast gapped-read alignment with Bowtie 2. *Nat Methods*. 2012;9:357–9.

23. Anders S, Pyl PT, Huber W. HTSeq-A Python framework to work with high-throughput sequencing data. *Bioinformatics*. 2015;31:166–9.

24. Stovner EB, Sætrom P. epic2 efficiently finds diffuse domains in ChIP-seq data. *Bioinformatics*. 2019;3–4.

25. Shao Z, Zhang Y, Yuan G-C, Orkin SH, Waxman DJ. MAnorm: a robust model for quantitative comparison of ChIP-Seq data sets. *Genome Biol*. 2012;13:R16.

26. Love MI, Huber W, Anders S. Moderated estimation of fold change and dispersion for RNA-seq data with DESeq2. *Genome Bio*l. 2014;15:550.

27. Li H, Durbin R. Fast and accurate short read alignment with Burrows-Wheeler transform. *Bioinformatics*. 2009;25:1754–60.

28. Thorvaldsdottir H, Robinson JT, Mesirov JP. Integrative Genomics Viewer (IGV): high-performance genomics data visualization and exploration. *Brief Bioinform*. 2013;14:178–92.

29. Zhang Y, Liu T, Meyer CA, Eeckhoute J, Johnson DS, Bernstein BE, et al. Model-based Analysis of ChIP-Seq (MACS). *Genome Biol*. 2008;9:R137.

30. Zang C, Schones DE, Zeng C, Cui K, Zhao K, Peng W. A clustering approach for identification of enriched domains from histone modification ChIP-Seq data. *Bioinformatics*. 2009;25:1952–8.

31. Hrdlickova R, Toloue M, Tian B. RNA-Seq methods for transcriptome analysis. Wiley Interdiscip Rev RNA. 2017 Jan;8(1). doi: 10.1002/wrna.1364.

32. Du Z, Zhou X, Ling Y, Zhang Z, Su Z. agriGO: A GO analysis toolkit for the

agricultural community. *Nucleic Acids Res*. 2010;38:64–70.

33. Supek F, Bošnjak M, Škunca N, Šmuc T. REVIGO summarizes and visualizes long lists of gene ontology terms. PLoS One. 2011;6(7):e21800. doi:10.1371/journal.pone.0021800.

34. Blümel M, Dally N, Jung C. Flowering time regulation in crops — what did we learn from Arabidopsis? *Curr Opin Biotechnol*. 2015;32:121–9.

35. Del Olmo I, Poza-Viejo L, Piñeiro M, Jarillo JA, Crevillén P. High ambient temperature leads to reduced FT expression and delayed flowering in Brassica rapa via a mechanism associated with H2A.Z dynamics. *Plant J*. 2019;0–3.

36. Theißen G, Rümpler F, Gramzow L. Array of MADS-Box Genes: Facilitator for Rapid Adaptation? *Trends Plant Sci*. 2018;23:563–76.

37. Goodrich J, Puangsomlee P, Martin M, Long D, Meyerowitz EM, Coupland G. A Polycomb-group gene regulates homeotic gene expression in Arabidopsis. *Nature*. 1997;386:44–51.

38. Chanvivattana Y, Bishopp A, Schubert D, Stock C, Moon Y-H, Sung ZR, et al. Interaction of Polycomb-group proteins controlling flowering in Arabidopsis. *Development.* 2004;131:5263–76.

39. Köhler C, Page DR, Gagliardini V, Grossniklaus U. The Arabidopsis thaliana MEDEA Polycomb group protein controls expression of PHERES1 by parental imprinting. *Nat Genet*. 2005;37:28–30.

40. Schubert D, Primavesi L, Bishopp A, Roberts G, Doonan J, Jenuwein T, et al. Silencing by plant Polycomb-group genes requires dispersed trimethylation of histone H3 at lysine 27. *EMBO J*. 2006;25:4638–49.

41. Huang Y, Liu C, Shen WH, Ruan Y. Phylogenetic analysis and classification of the Brassica rapa SET-domain protein family. *BMC Plant Biol*. 2011;11.

42. Stephenson P, Baker D, Girin T, Perez A, Amoah S, King GJ, et al. A rich TILLING resource for studying gene function in Brassica rapa. *BMC Plant Biol*. 2010;10:62.

43. Serrano-Cartagena J, Candela H, Robles P, Ponce MR, Perez-Perez JM, Piqueras

P, et al. Genetic analysis of incurvata mutants reveals three independent genetic operations at work in Arabidopsis leaf morphogenesis. *Genetics*. 2000;156:1363–77.

44. Saha G, Park J-I, Jung H-J, Ahmed NU, Kayum MA, Chung M-Y, et al. Genome-wide identification and characterization of MADS-box family genes related to organ development and stress resistance in Brassica rapa. *BMC Genomics*. 2015;16:178.

45. Cheng F, Liu S, Wu J, Fang L, Sun S, Liu B, et al. BRAD, the genetics and genomics database for Brassica plants. *BMC Plant Biol*. 2011;11:136–41.

46. Springer NM. Epigenetics and crop improvement. *Trends Genet.* 2012;29:241–7.

47. Gallusci P, Dai C, Génard M, Gauffretau A, Leblanc-Fournier N, Richard-Molard C, et al. Epigenetics for Plant Improvement: Current Knowledge and Modeling Avenues. *Trends Plant Sci*. 2017;22:610–23.

48. Poza-Viejo L, Del Olmo I, Crevillén P. Plant Chromatin Immunoprecipitation V.2. *protocols.io*. 2019. https://dx.doi.org/10.17504/protocols.io.zmff43n

49. Xu X, Yang Z, Sun X, Zhang L, Fang Z. Selection of reference genes for quantitative real-time PCR during flower bud development in CMS7311 of heading Chinese cabbage (Brassica rapa L. ssp. pekinensis). *Acta Physiol Plant*. 2014;36:809–14.

50. Jaitin DA, Kenigsber E, Keren-Shaul H, Elefant N, Paul F, Zaretsky I et al. Massively parallel single-cell RNA-seq for marker-free decomposition of tissues into cell types. *Science*. 2014;343:776LP-779.

51. Meinhof K, Leader A, Kobayashi S, Remark R, Rytlewski JA, Beasley MB, et al. Innate Immune Landscape in Early Lung Adenocarcinoma by Paired Single-Cell Analyses. *Cell*. 2017;169:750-765.e17.

52. Bolger AM, Lohse M, Usadel B. Trimmomatic: a flexible trimmer for Illumina sequence data. *Bioinformatics*. 2014;30:2114–20.

53. Li H, Handsaker B, Wysoker A, Fennell T, Ruan J, Homer N, et al. The Sequence Alignment/Map format and SAMtools. *Bioinformatics*. 2009;25:2078–9.

54. Picard tools. Broad Institute. *GitHub Repository*. 2018.

https://github.com/broadinstitute/picard
55. Ewels P, Magnusson M, Lundin S, Käller M. MultiQC: summarize analysis results for multiple tools and samples in a single report. *Bioinformatics*. 2016;32:3047–8.
56. Ramírez F, Ryan DP, Grüning B, Bhardwaj V, Kilpert F, Richter AS, et al. deepTools2: a next generation web server for deep-sequencing data analysis. *Nucleic Acids Res*. 2016;44:W160–5.
57. Zhu LJ, Gazin C, Lawson ND, Pagès H, Lin SM, Lapointe DS, et al. ChIPpeakAnno: a Bioconductor package to annotate ChIP-seq and ChIP-chip data. *BMC Bioinformatics*. 2010;11:237.
58. Shen L, Shao N, Liu X, Nestler E. ngs.plot: Quick mining and visualization of next-generation sequencing data by integrating genomic databases. *BMC Genomics*. 2014;15:284.
59. Martin PG. Nicer scatter plots in GGgally ggpairs-ggduo. 2018. http://genoweb.toulouse.inra.fr/~pmartin/pgpmartin/2018/11/14/nicer-scatterplot-in-gggally/
60. Stanke M, Schöffmann O, Morgenstern B, Waack S. Gene prediction in eukaryotes with a generalized hidden Markov model that uses hints from external sources. *BMC Bioinformatics*. 2006;7:62.
61. Miriam Payá Milans, Mark Wilkinson, Laura Poza-Viejo, Patxi San Martín-Uriz, David Lara-Astiaso, Pedro Crevillén. 2019. wilkinsonlab/epigenomics_pipeline: Epigenomics pipeline for Brassica data analysis. doi:10.5281/zenodo.3298029.
62. Payá-Milans M; Poza-Viejo L; Martín-Uriz PS; Lara-Astiaso D; Wilkinson MD; Crevillén P (2019): Supporting data for "Genome-wide analysis of the H3K27me3 epigenome and transcriptome in Brassica rapa" GigaScience Database. http://dx.doi.org/10.5524/100672

Click here to access/download;Figure;Figures r1 1.pdf

Figure 1

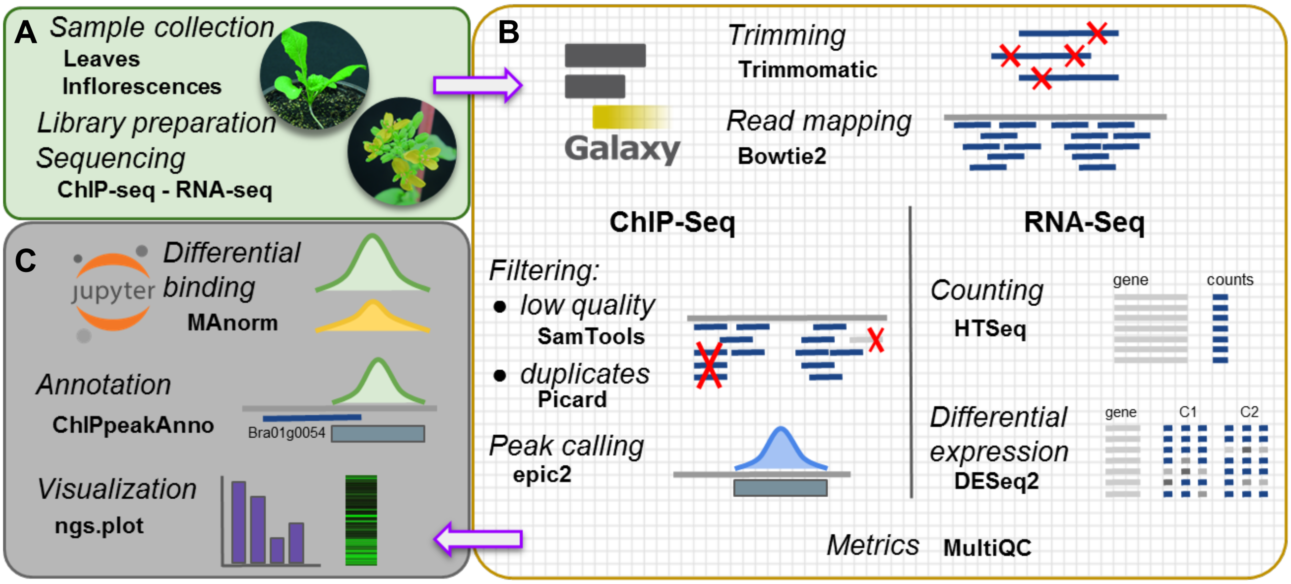

Figure 2

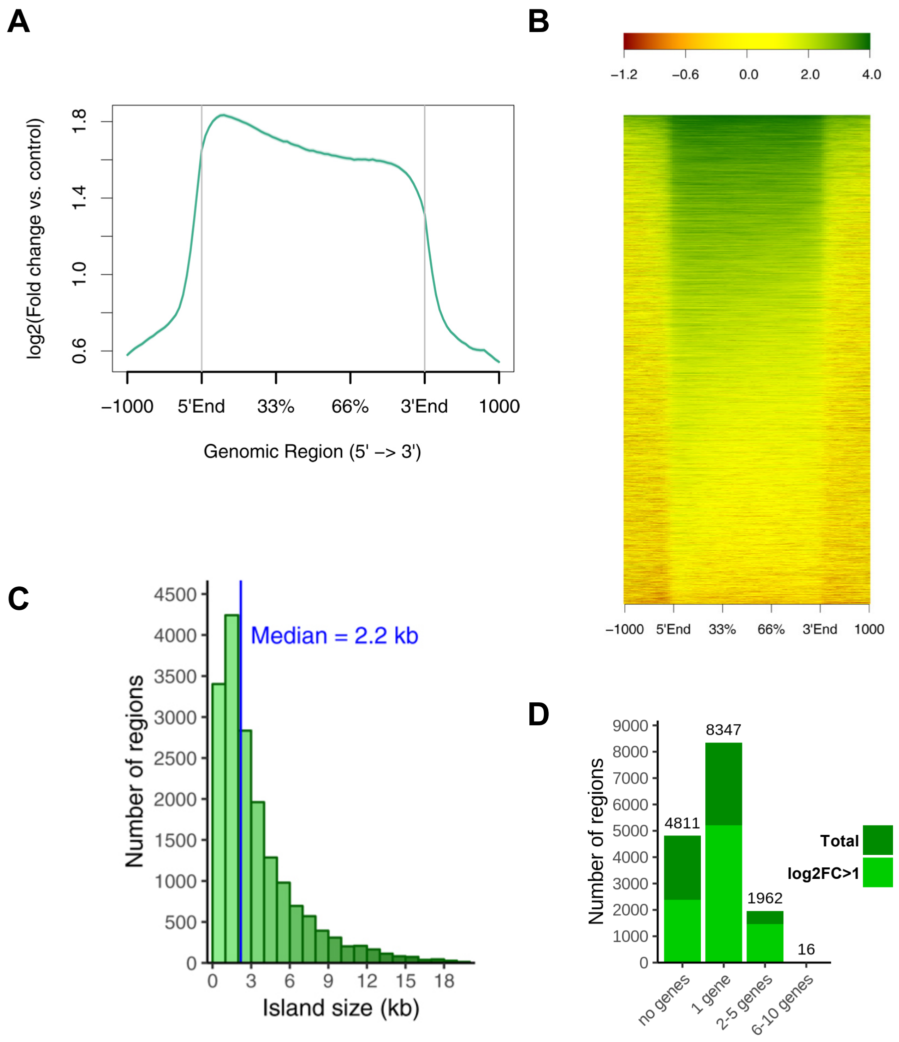
A
log2(Fold change vs. control)
1.8
1.4
1.0
0.6
−1000
5'End
33%
66%
3'End
1000
Genomic Region (5' –> 3')
B
−1.2
−0.6
0.0
2.0
4.0
−1000
5'End
33%
66%
3'End
1000
C
Number of regions
4500
4000
3500
3000
2500
2000
1500
1000
500
0
Median = 2.2 kb
0
3
6
9
12
15
18
Island size (kb)
D
Number of regions
9000
8000
7000
6000
5000
4000
3000
2000
1000
0
4811
8347
1962
16
no genes
1 gene
2-5 genes
6-10 genes
Total
log2FC>1

Click here to access/download;Figure;Figures r1 3.pdf

Figure 3

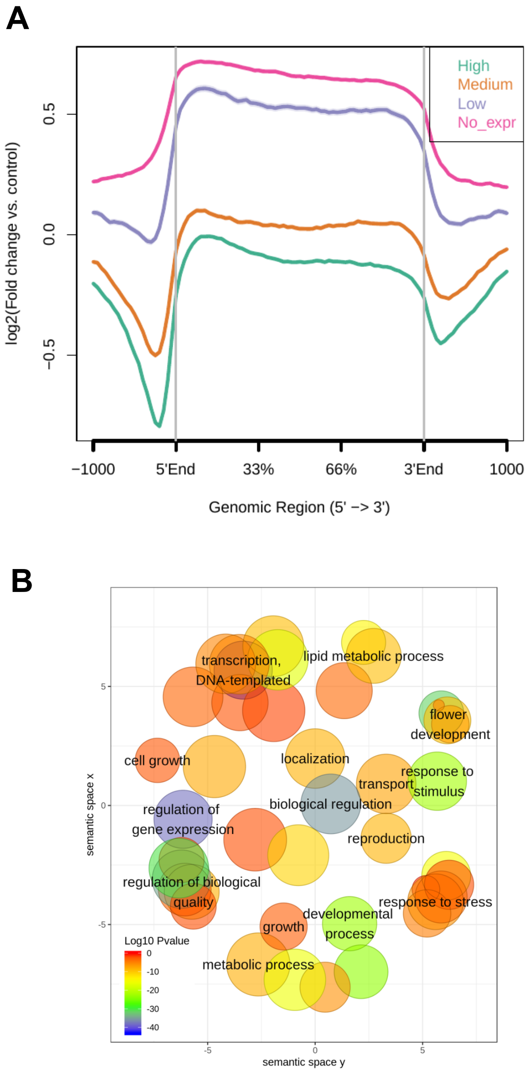

Click here to access/download;Figure;Figures r1 4.pdf

Figure 4

A

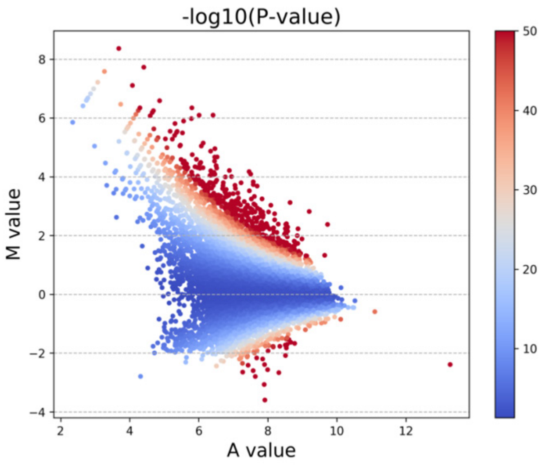

B

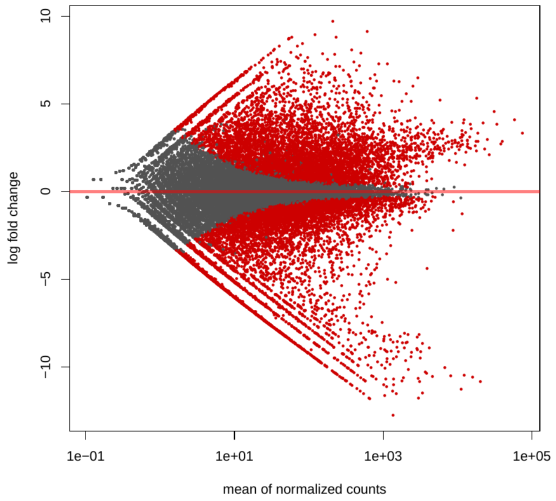

C

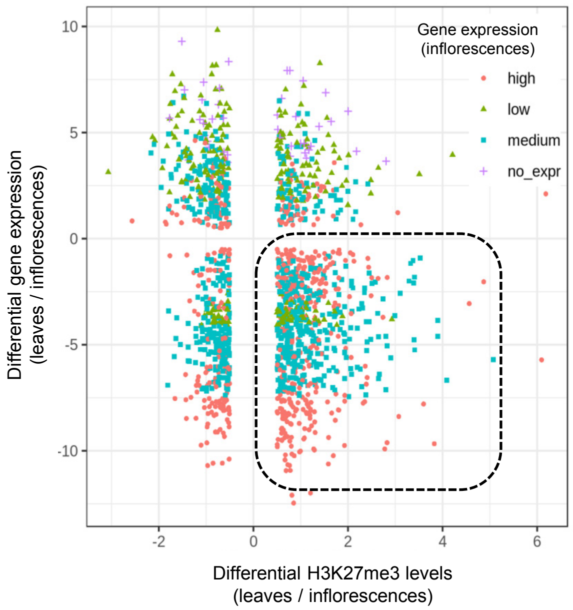

D

Accession	Term	FDR
GO:0048438	floral whorl development	1E-15
GO:0006357	regulation of transcription	1.6E-14
GO:0009908	flower development	1.6E-14
GO:0048437	floral organ development	2.2E-14
GO:0090567	reproductive shoot system development	2.2E-14
GO:0006366	transcription from RNA polymerase II promoter	1.5E-13
GO:2001141	regulation of RNA biosynthetic process	4.1E-13
GO:0006355	regulation of transcription, DNA-templated	4.1E-13
GO:1903506	regulation of nucleic acid-templated transcription	4.1E-13
GO:0019219	regulation of nucleobase-containing compound metabolic process	8.8E-13

 Click here to access/download;Figure;Figures r1 5.pdf

Figure 5

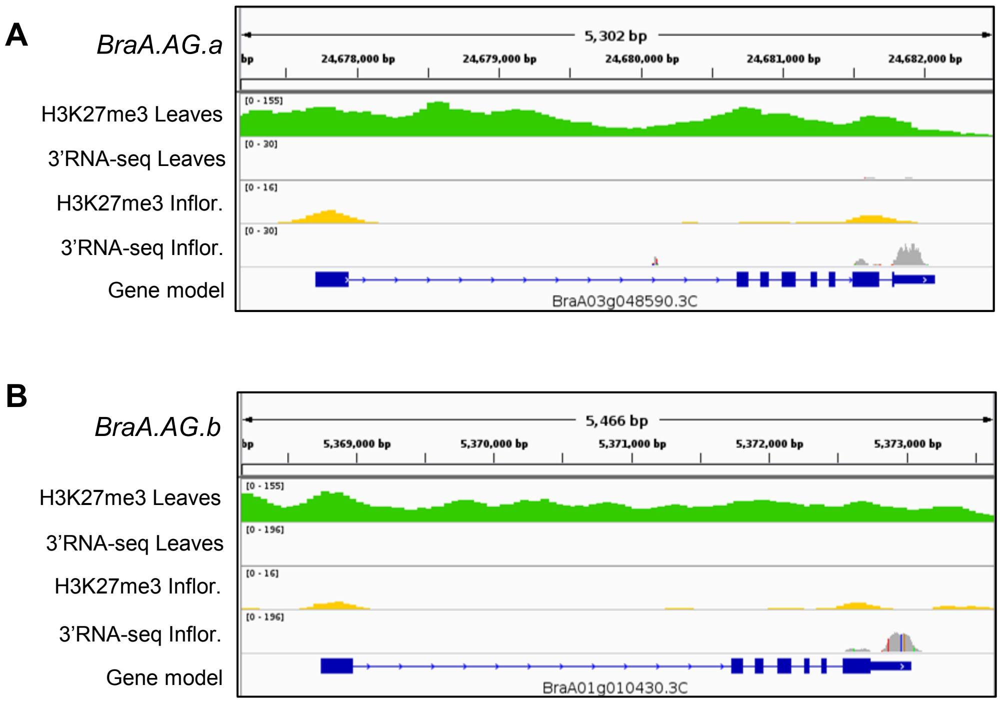

Click here to access/download;Figure;Figures r1 6.pdf

Figure 6

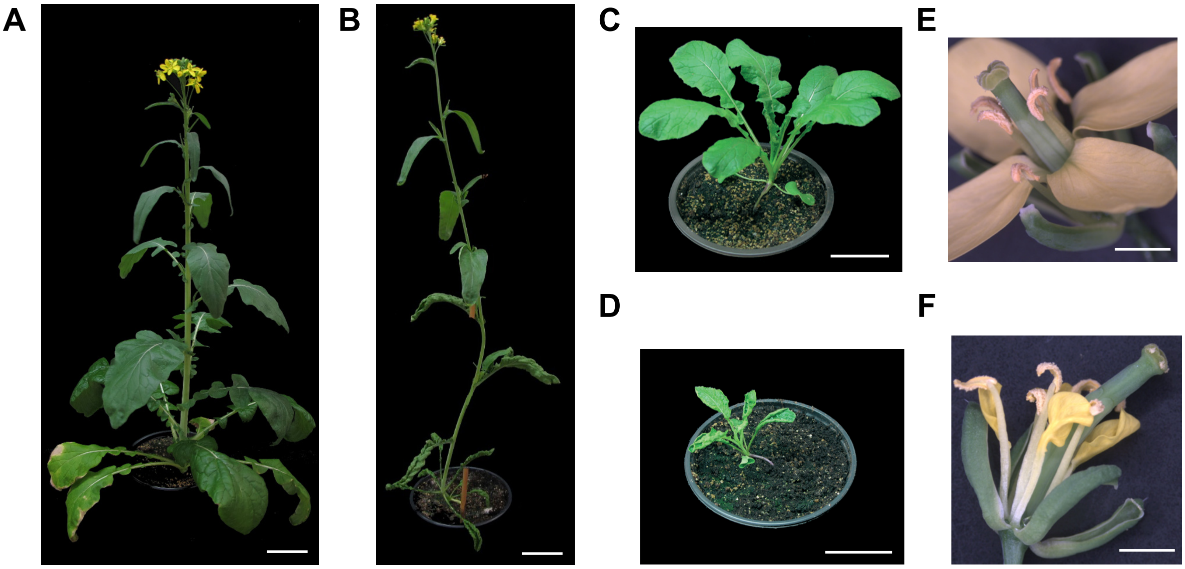

Figure 7

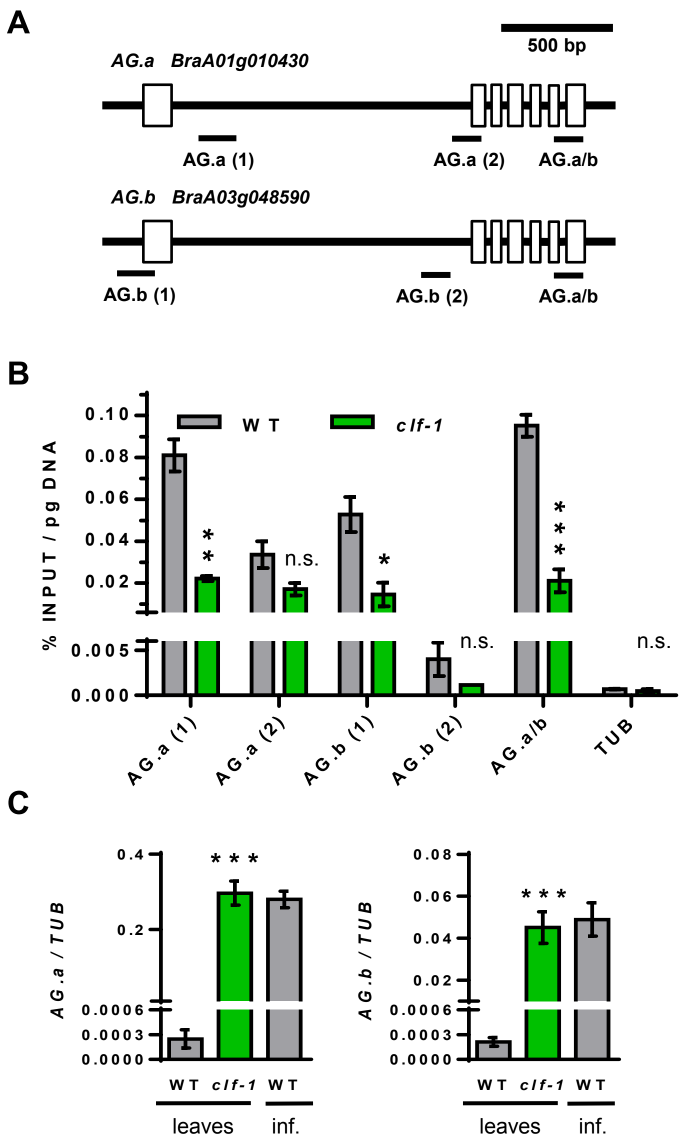
A
500 bp
AG.a BraA01g010430
AG.a (1)
AG.a (2)
AG.a/b
AG.b BraA03g048590
AG.b (1)
AG.b (2)
AG.a/b
B
WT
clf-1
% INPUT / pg DNA
0.10
0.08
0.06
0.04
0.02
0.005
0.000
n.s.
AG.a (1)
AG.a (2)
AG.b (1)
AG.b (2)
AG.a/b
TUB
C
AG.a / TUB
0.4
0.2
0.0006
0.0003
0.0000
WT clf-1 WT
leaves
inf.
AG.b / TUB
0.08
0.06
0.04
0.02
0.0006
0.0003
0.0000
WT clf-1 WT
leaves
inf.

Additional File 1

Click here to access/download
Supplementary Material
Additional File 1 r1.pdf

Additional File 2

Click here to access/download
Supplementary Material
Additional File 2r1.docx

Additional File 3

Click here to access/download
Supplementary Material
Additional file 3r1.xlsx
