# Supplementary material for: Genome-wide analysis of the H3K27me3 epigenome and transcriptome in Brassica rapa
Source: Gigascience. 2019 Dec 4;8(12):giz147. doi: 10.1093/gigascience/giz147 (PMC6892454; doi:10.1093/gigascience/giz147)
Supplement: giz147_Supplemental_Files [file giz147_supplemental_files.zip › Additional File 2r1.docx]

**Table S1.** **Alignment statistics**

| **Sample** | **Total reads** | **Reads processed^1^** | **Reads mapped** | **Reads duplicated^2^** | **Multi-mapped reads^3^** | **Unmapped reads** |
| --- | --- | --- | --- | --- | --- | --- |
| **ChIP-seq data** |  |  |  |  |  |  |
| ChIP leaves 1 | 34 221 777 | 33 944 048 | 27 927 380  (82.27%) | 2 057 998 | 13 677 527  (40.29%) | 6 016 668  (21.54%) |
| ChIP leaves 2 | 33 607 646 | 33 332 369 | 26 167 326  (78.50%) | 3 164 609 | 10 888 647  (32.67%) | 7 165 043  (27.38%) |
| Input leaves 1 | 30 100 210 | 29 830 222 | 25 839 673  (86.62%) | 1 441 515 | 15 772 577  (52.87%) | 3 990 549  (15.44%) |
| Input leaves 2 | 32 128 301 | 31 840 333 | 27 401 947  (86.06%) | 1 953 458 | 15 954 670  (50.11%) | 4 438 386  (16.20%) |
| ChIP Inflor. | 52 487 139 | 51 188 308 | 38 032 966  (74.30%) | 15 223 233 | 25 723 267  (50.25%) | 13 155 342  (34.59%) |
| Input Inflor. | 42 687 680 | 41 520 400 | 34 331 529  (82.69%) | 9 732 840 | 24 179 801  (28.24%) | 7 188 871  (20.94%) |
| **RNA-seq data** |  |  |  |  |  |  |
| RNA leaves 1 | 6 772 532 | 6 637 268 | 5 743 832  (86.54%) |  | 2 584 906  (38.95%) | 893 436  (15.55%) |
| RNA leaves 2 | 12 919 136 | 12 641 420 | 10 886 556  (86.12%) |  | 4 678 211  (37.01%) | 1 754 864  (16.12%) |
| RNA leaves 3 | 15 591 489 | 15 309 096 | 13 148 531  (85.89%) |  | 5 722 760  (37.38%) | 2 160 565  (16.43%) |
| RNA Inflor. 1 | 11 720 585 | 11 436 845 | 8 859 097  (77.46%) |  | 2 520 001  (22.03%) | 2 577 748  (29.10%) |
| RNA Inflor. 2 | 11 620 362 | 11 311 764 | 8 771 920  (77.55%) |  | 2 520 001  (22.28%) | 2 539 844  (28.95%) |
| RNA Inflor. 3 | 17 056 472 | 16 642 015 | 12 834 173  (77.12%) |  | 3 439 787  (20.67%) | 3 807 842  (29.67%) |

^1^ Removed adapter sequences, bases with quality < 15 and reads with length < 20 bp.

^2^ Mapping and optical duplicates were marked.

^3^ The highest scoring alignment was kept.

**Table S2. Comparison of Bowtie2 and BWA performance.**

|  | **Bowtie2^1^** | **BWA** |
| --- | --- | --- |
| Total reads | 91,003,820 | 91,003,820 |
| Mapped reads | 69,808,788  (76.71%) | 74,545,634  (81.91%) |
| Reads properly paired | 69,808,788  (76.71%) | 68,597,753  (75.38%) |
| Singletons | 0  (0%) | 1,781,047  (1.96%) |
| Mate on different chromosome | 0  (0%) | 2,850,905  (3.13%) |

^1^ No-mixed and no-discordant options prevent non concordant alignments

**Table S3**. **H3K27me3 regions identified in *B. rapa*.**

|  |  |  | **Leaves** | **Inflorescences** |
| --- | --- | --- | --- | --- |
| **A**  # peaks | | | 15 136 | 20 967 |
| # bases | | | 45 264 600 | 43 262 800 |
| genome coverage | | | 12.89 % | 12.32 % |
| median peak lenght | | | 2.2 kb | 1.6 kb |
| avg. peak lenght | | | 2.9 kb | 2.0 kb |
| shortest peak | | | 0.2 kb | 0.2 kb |
| longest peak | | | 43.8 kb | 10.0 kb |
| **B** # intergenic peaks | | | 4 811 | 10 787 |
|  | # peaks on genes^1^ | | 10 325 | 10 180 |
|  | 1 gene | | 8 347 | 9 137 |
|  |  | 2-5 genes | 1 962 | 1 038 |
|  |  | >5 genes | 16 | 5 |
|  | # marked genes |  | 12 480 | 10 807 |
|  | # peak-gene intersections | | 12 949 | 11 395 |

^1^ Marked genes are defined as those that have at least 1 bp overlapping with an H3K27me3 peak.
