# Supplementary figures and images for: Genome-wide analysis of the H3K27me3 epigenome and transcriptome in Brassica rapa
Source: Gigascience. 2019 Dec 4;8(12):giz147. doi: 10.1093/gigascience/giz147 (PMC6892454; doi:10.1093/gigascience/giz147)

Figure S1

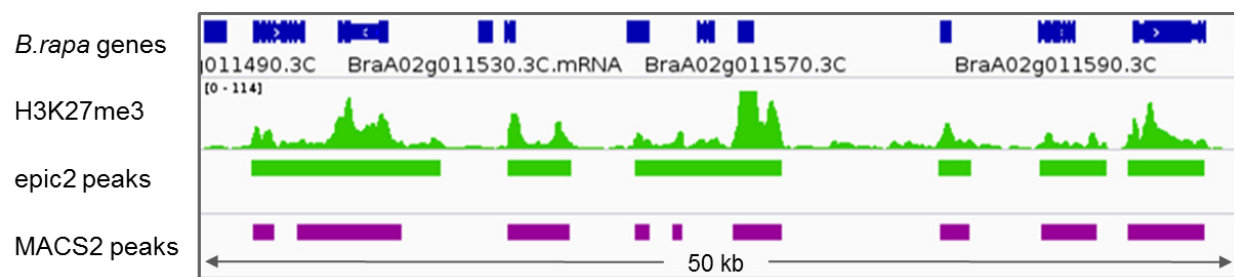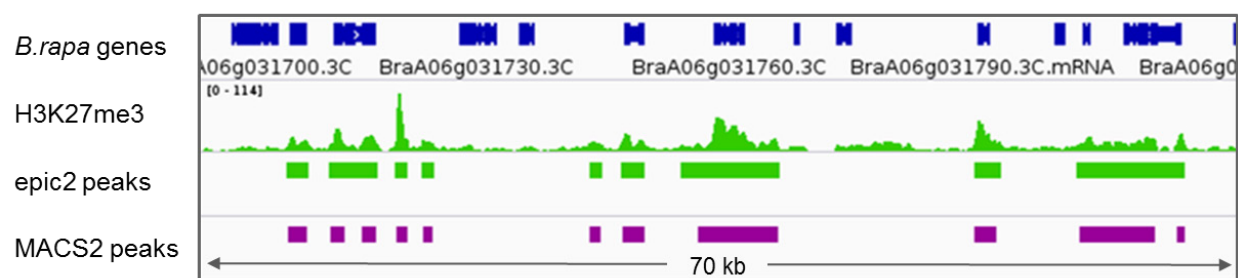

Figure S2

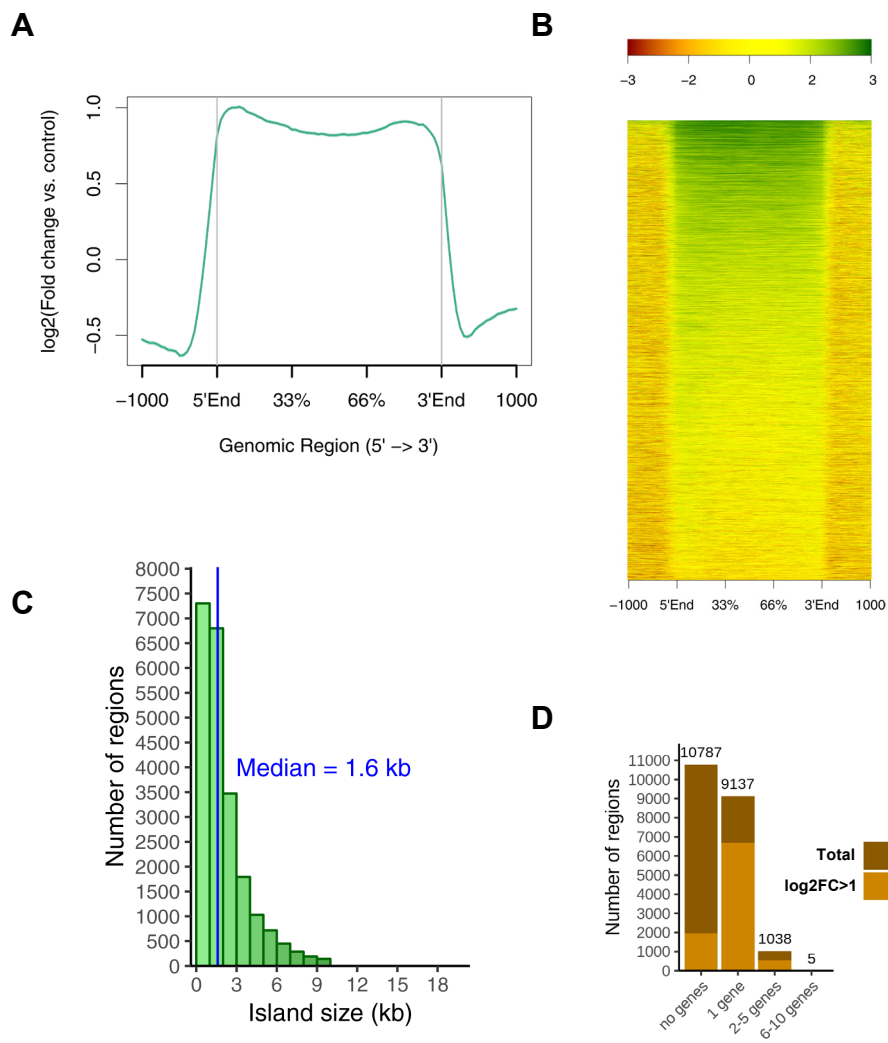

Figure S3

A

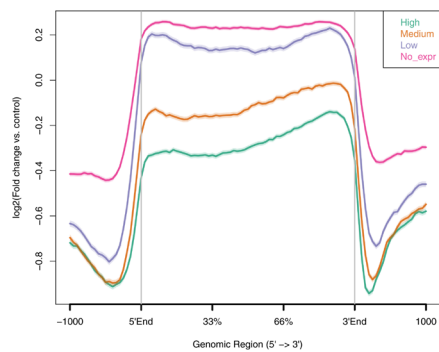

B

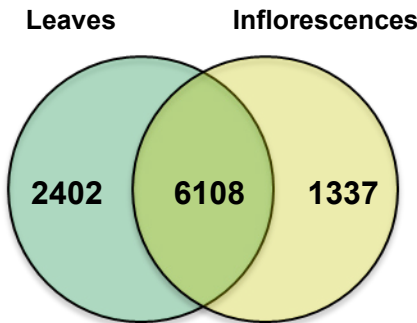

C

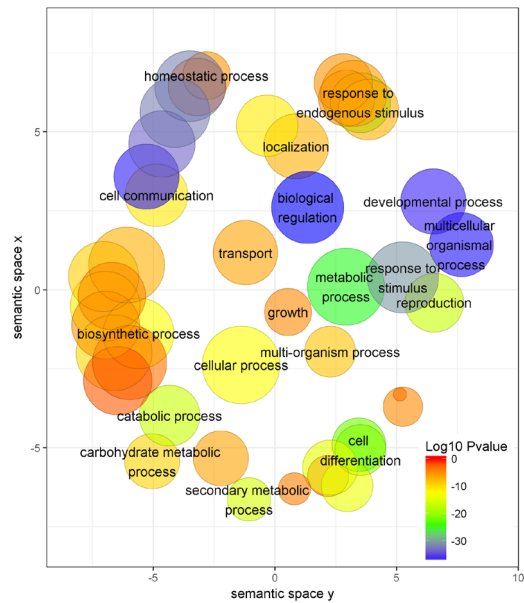

Figure S4

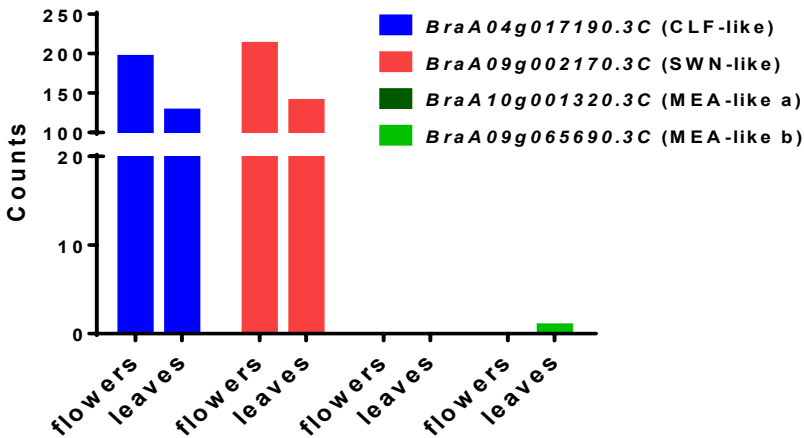

Figure S5

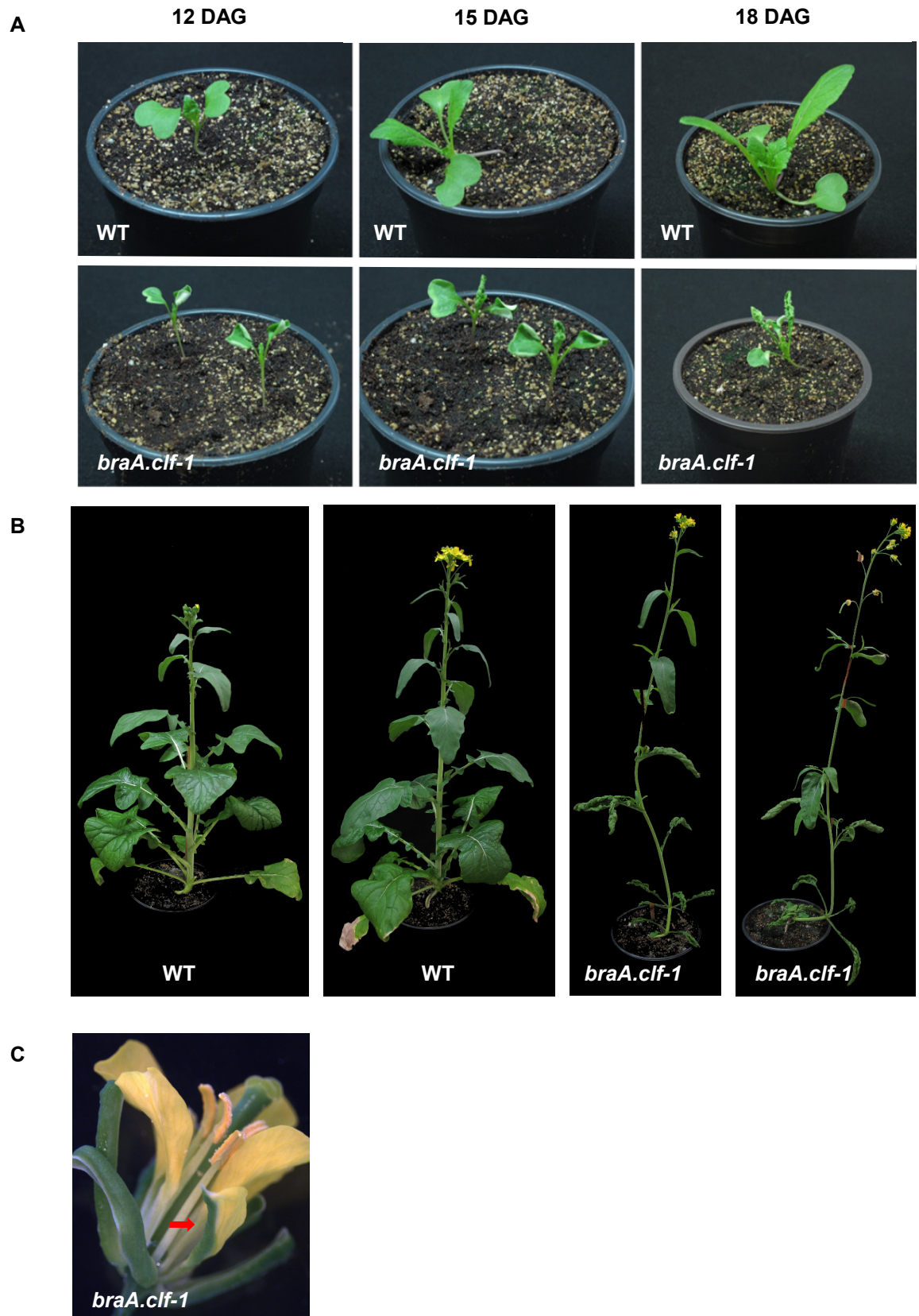

Figure S6

**A**

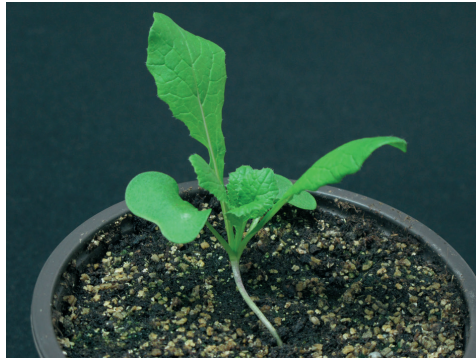

**B**

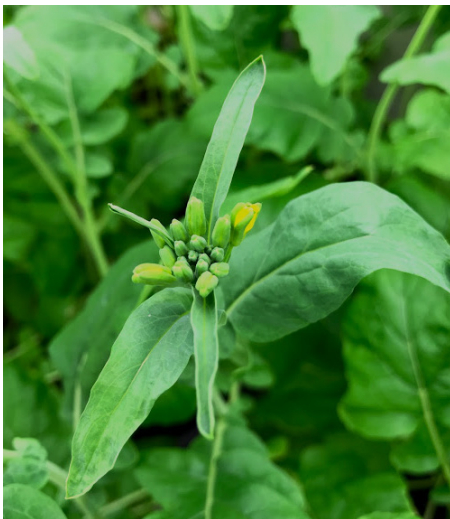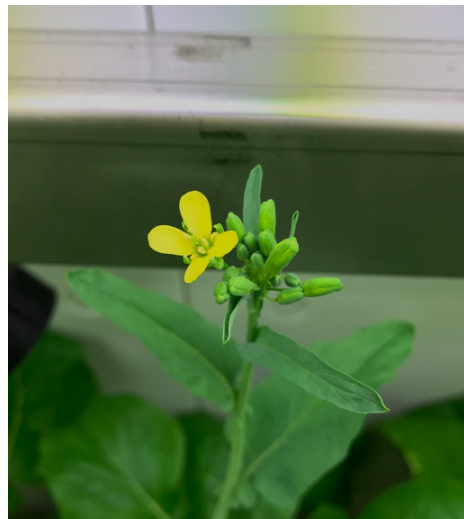

Figure S7

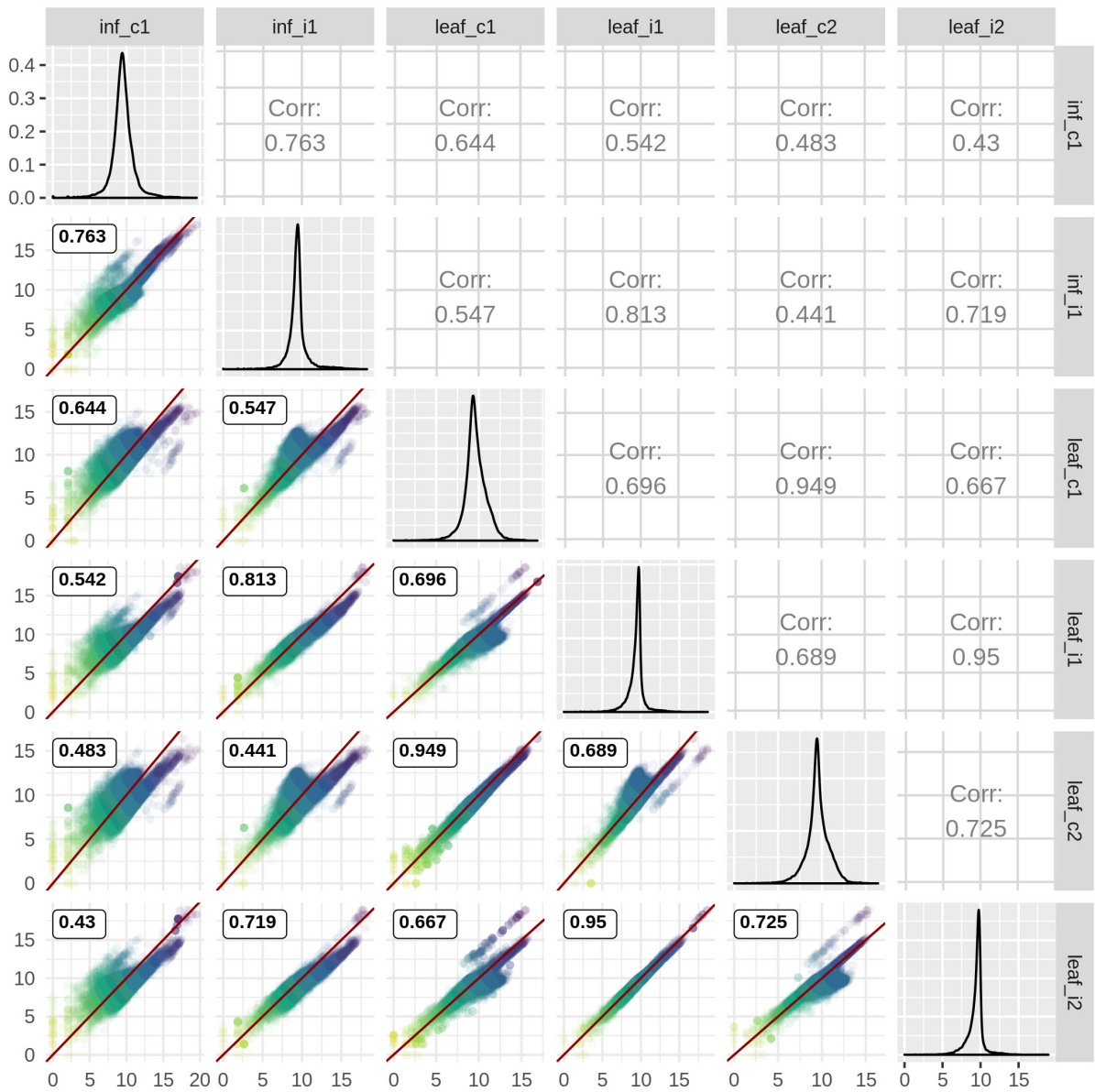

Figure S8

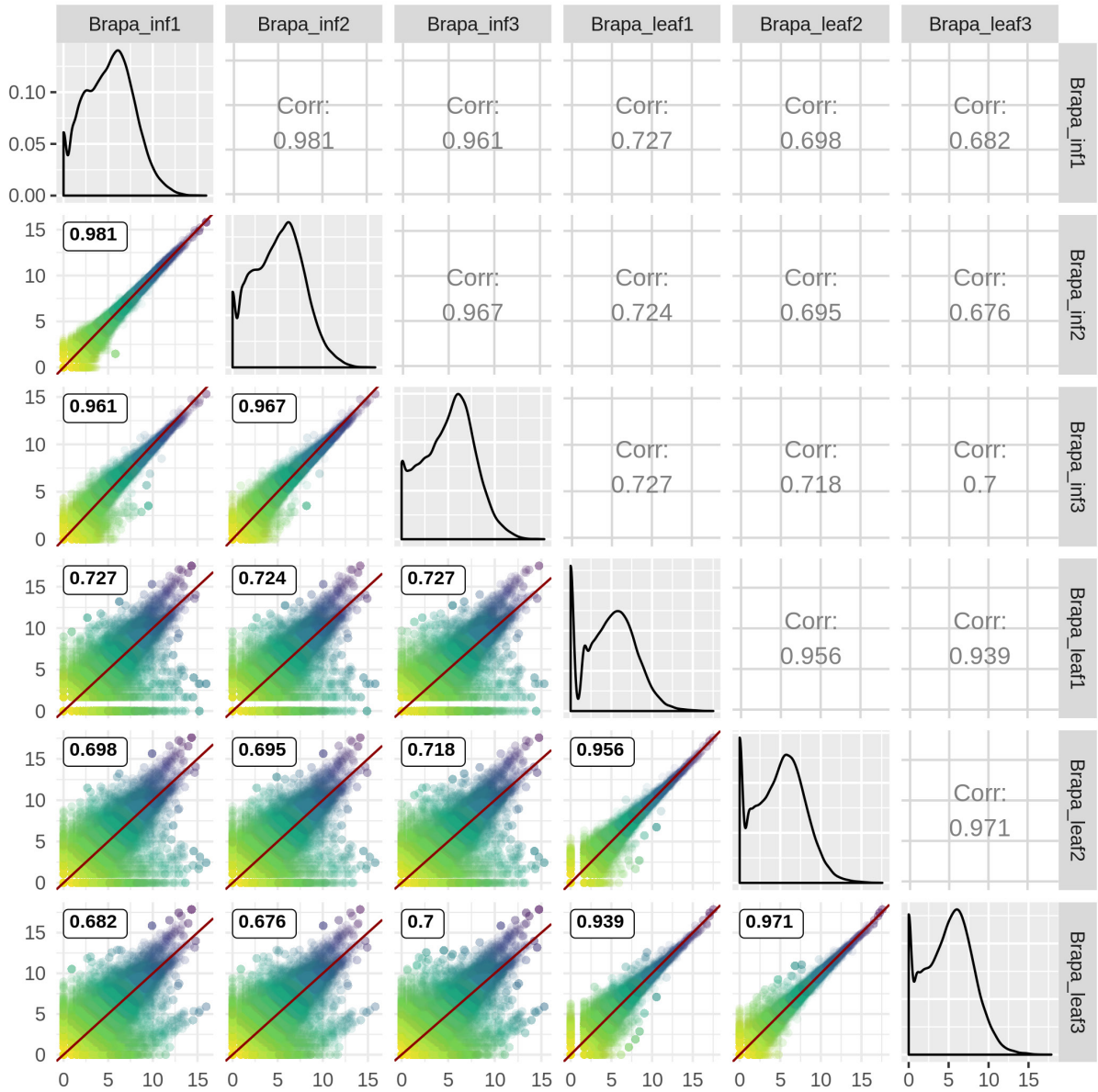

Supplement: giz147_Supplemental_Files [file giz147_supplemental_files.zip › Additional File 1 r1.pdf]
