# Supplementary material for: Genome-wide analysis of the H3K27me3 epigenome and transcriptome in Brassica rapa
Source: Gigascience. 2019 Dec 4;8(12):giz147. doi: 10.1093/gigascience/giz147 (PMC6892454; doi:10.1093/gigascience/giz147)
Supplement: giz147_Response_to_Reviewer_Comments_Original_Submission [file giz147_response_to_reviewer_comments_original_submission.pdf]

Author's Response To Reviewer Comments

Close

RESPONSE TO REVIEWER COMMENTS:

We are grateful for all the comments of both reviewers. Following reviewer's suggestions some parts of the results and methods has been edited. This has improved the paper although our main conclusions remain the same. In this revised version we added new inflorescences ChIP-seq and RNA-seq analyses (Fig. S2 and S3, and table S3), new IGV snapshots of key developmental genes (Fig. 5), new statistical analyses of ChIP-qPCR data (Fig.7), new FigS5 with more representative mutant plant pictures (Fig. S5) and new correlation analyses between replicate genomic samples (Fig.S7 and S8). As suggested, we also registered our pipeline at SciCrunch and bio.tools databases.

We answered all questions and made some clarifications along the text.

Reviewer #1

The authors characterise the distribution of H3K27me3 across B. rapa leaves and inflorescence tissues, alongside its importance for appropriate development and flowering through gene silencing. Overall, the data appears to be solid providing a resource for the community and extends known relationships into new species. I highly commend the authors on the development and execution of the REA pipeline (aligning with FAIR principles), and hope this helps to establish a new "precedent" for increasing accessibility and rigour in our data & analyses.

RESPONSE: We are glad that reviewer #1 expressed such enthusiasm for the development and execution of our bioinformatic pipeline. We share the same hope for reproducibility and rigour.

I have some concerns/requests, which I think will strengthen the paper prior to publication:

1. While this study characterises the distribution of H3K27me3, it is not exploring anything epigenetic. Instead, this reference to epigenetics is reinforcing the vague use of the term, which is what the authors in ref 1 argue against. I recommend the first sentence should be deleted and all references to epigenetic(s) should instead refer to "histone modification(s)", "chromatin mark(s)" or "epigenome", where the latter is clearly defined.
RESPONSE: We see the point of the reviewer #1; the first sentence of the manuscript was deleted and reference 1 modified accordingly.

2. Could the first use of "BraA.CLF" include the full name (i.e. define the abbreviation). RESPONSE: The text in the last paragraph of the introduction has been edited.

3. Could the authors please clarify whether "from the same plant samples" (p. 7) means from the same tissues but independent plants or aliquots of the same harvested tissue?
RESPONSE: We took samples for RNA and ChIP from the same harvested plants. The text has been edited in Data Description and Methods sections to clarify this point.

4. P. 8 Please include an appropriate citation for the sentence starting with "A metagene plot of H3K27me3 ... as described in other plant species, ..." for clarity. RESPONSE: We added the proper citations.

5. For Fig 2A, can the signal from leaf vs inflorescence tissue be plotted separately? Could some measure of variance (standard deviation or standard error) be included in the plots?
RESPONSE: In the Figure 2, only leaves data was shown as representative for the H3K27me3 mark. Following both reviewers suggestions a new Fig. S2 and S3 including the analysis of the Inflorescence ChIP signal has been added. About Fig. 2A, we redid the metagene plots using ngs.plot including the standard error, but the deviation is so small that it is barely visible in the figure.

6. Fig 2C, can the authors please mark the median. I find this more informative than the mean for this type of data.
RESPONSE: As suggested, mean value has been substituted by median in the new Fig2C.

7. P. 9, I am a bit concerned about the one-fold increase threshold used for ChIP signal. This threshold seems too low to reduce background noise and the analysis may benefit from using a threshold FC>1.2 - 1.5x (i.e. 20-50% increase from background). RESPONSE: We apologize for the mistake, but we meant Log2FoldChange which is the output of epic2. Thus a log2FC>1 indicates over a 100% increase from background. We corrected this nomenclature over the paper, and including log2FC>1 number of peaks in new Fig. 2D and S2D.

8. Could the authors specify "H3K27me3" throughout manuscript. There are some references to "H3K27" or "H3K27 methylation", which should be replaced.
RESPONSE: We edited the text as suggested.

9. Could the authors please make Fig S2 more presentable. This could probably be combined with Fig S1.
RESPONSE: We have redrawn the Venn diagram and included it in the new Fig. S3 together with new ChIP-seq inflorescences analyses.

10. Fig 4C - If high/med/low expression levels were based on inflorescence tissue, I would have expected more "lowly" expressed genes in the explored quadrant (down-regulated genes with increased H3K27me3). Instead there are many med-highly expressed genes in the "down-regulated" portion of the figure. There are also a number of "no_expr" in the up-regulated gene set. I am unsure how to reconcile this except to ask the authors to reproduce this figure/analysis (e.g. is it possible up- and down-regulated genes were switched or the contrast performed was relative to leaf instead of inflorescence?).
RESPONSE: We think that reviewer #1 misunderstood the performed contrast. In the Fig. 4C, the expression and H3K27me3 changes are relative to leaves. To make it clear we edited figure axes and the text along the results to help the reader.

The p.adj-cutoff used here also seems higher than the commonly, yet arbitrary, used levels of 0.05 or 0.01. I would also appreciate any comments from the authors on this.
RESPONSE: This work was aimed to investigate broad patterns of expression combined with histone marking, and to capture higher diversity we selected differentially expressed genes with padj<0.1, which is still considered statistically significant. However, we see the point of the review and to be consistent throughout the text we edited the number of reported DEG genes to indicate 14,697 DEG p-adj< 0.1 instead of 13,377 DEG p-adj< 0.05.

11. Can the authors please clarify the 1,724 overlap of genes with changed H3K27me3 and mRNA levels. Were the 4,729 deferential H3K27me3 genes overlapped with 13,377 DEGs to give 1,724 genes? Please also perform a Fisher's exact test for some level of statistical confidence.
RESPONSE: The 1,724 genes referred to the genes both marked and differentially expressed (|M/log2FC|>0.5 and padj<0.1; filtering setting on Github). Nonetheless, we found that this sentence is also confusing and does not add any biological information, so we deleted and edited the text accordingly in the new version of the manuscript.
We focused on the 729 loci that showed reduced H3K27me3 and increased expression from leaves to inflorescences. This result is enriched 2.07 fold compared to expectations (hypergeometric tests; p-value = 2.9e-164).

12. P.12 sentence beginning with "All these developmental abnormalities ..." - please include citation for clarity.
RESPONSE: This part of the text was edited for clarification.

13. A browser shot for H3K27me3 and mRNA at the B. rapa AG loci would be nice.
RESPONSE: Thanks for the suggestion, we added a new Fig. 5 with this data.

14. Please specify ChIP qPCR p. 12 sentence beginning with "We performed ChIP experiments...".
RESPONSE: The text was edited for clarification.

15. An ANOVA with post-hoc tukey tests should be performed for Fig 6 B-C.
RESPONSE: We added statistical tests to all the data in the new Fig. 7. We also redraw figure B to put all ChIP-qPCR under the same scale to clarify the difference between H3K27me3 marked loci and Tubulin

locus.

16. Were braA.clf-1 seeds ensured to be homozygous?
RESPONSE: We always used homozygous mutant plants genotyped by PCR and sequencing. We edited the methods to clarify this point.

17. Was RNA integrity checked prior to qPCR and 3' RNA sequencing?
RESPONSE: As a standard routine in the laboratory we always check RNA integrity by agarose gel electrophoresis. In addition for genomic experiments RNA, integrity was determined using a TapeStation system. This information has been added to the manuscript.

18. If possible, could the authors re-analyse their raw qPCR fluorescence data using LinReg PCR (Ramakers et al 2003, Ruijter et al 2009).
RESPONSE: We are unsure of the utility of this suggestion. In our experience LinReg PCR software is not totally reliable. For all calculations we determined the threshold cycle (CT) and used the "2-ΔΔCT method", which is a widely accepted method for qPCR quantification. However, before using any primer we tested the primer amplification efficiency by doing qPCR reactions of serial dilutions of the target amplicon. All our primers showed amplification between 85-115%. We add this information into the manuscript.

19. P. 17, "A first step of trimming was performed with [52] v0.36.5 ." should be "A first step of trimming was performed with Trimmomatic (v0.36.5) [52].
RESPONSE: We edited the text as suggested.

20. Fig S4: could the authors please clarify how many flowers were tested for WT and clf-1?
RESPONSE: We dissected under the microscope the apical flower from the main inflorescence of 20 mutant plants and found 1 flower with homeotic transformations. We never observed this phenotype in the wild-type plant (>100 flowers). We add this information to the new Fig5S legend. We understand that this number may not be statistically significant, thus we edited the text accordingly. In any case, we believe this information is important to the reader as it is remarkably similar to the Arabidopsis clf mutant phenotype.

Reviewer #2

In this MS, the author demonstrates the profile of H3K27me3 in different organ of Brassica rapa, and uncover its role in plant development with mutant analysis. Though the analysis of H3K27me3 has been done in B. rapa before, it focused only on specific region (Genes Genet Syst. 2016, 91:1-10). This study provides a genomic-wide view of this type of histone modification, enable us to get a comprehensive understanding of its function.
There are also some suggestions listed as below:

1. For high-throughput data, three independent biological repeats are required. Although the author demonstrates the reproducibility of the Epigenomic Analysis pipeline, please make it clear whether the replicate tests has been performed to ensure the accuracy of the results.
RESPONSE: We agree with reviewer #2 that biological repeats are important in genomic analyses. In fact, we analyzed three biological replicates for all RNA-seq experiments. ChIP-seq signal is not as variable as RNA expression and less replicates are usually performed. Thus, two biological duplicates for the leaf H3K27me3 ChIP-seq were used. However, in the case of the inflorescence H3K27me3 ChIP-seq data we only studied one biological replicate because this is an heterogeneous material (young and old flowers are mixed). We understand that for the purpose of this study one replicate is enough, because we used inflorescence ChIP-seq data to identify remarkable differences between the two studied organs. In addition, following reviewer #2 suggestions, we have made new replicate test between our samples that are included in a new Fig. S7 and S8.

2. The H3K27me3 modification analysis has been done in both leaves and inflorescences. The Chip-seq data for inflorescences also deserves a figure to show its details.
RESPONSE: We thank reviewer #2 suggestions. Inflorescence ChIP analysis has been added added into the new Fig.S2 and S3.

3. For figure 5, the mutant and the wild type are in different pictures with different bars. If they are in the same picture, it would be much easier for the comparison. At least their bars should be the same.
RESPONSE: Following reviewer #2 suggestion we have edited Fig. 5 to make easier the comparison

between wild-type and mutant plants. To aid the reader, we add more plant pictures in the new Fig.S5.
Please note that all pictures are from plants grown together.

Close
