# Supplementary material for: Genome-wide analysis of the H3K27me3 epigenome and transcriptome in Brassica rapa
Source: Gigascience. 2019 Dec 4;8(12):giz147. doi: 10.1093/gigascience/giz147 (PMC6892454; doi:10.1093/gigascience/giz147)
Supplement: giz147_Reviewer_1_Report_Original_Submission — Diep Ganguly -- 8/28/2019 Reviewed [file giz147_reviewer_1_report_original_submission.pdf]

Reviewer Report

Title: Genome-wide analysis of the H3K27me3 epigenome and transcriptome in Brassica rapa

Version: Original Submission **Date:** 8/28/2019

Reviewer name: Diep Ganguly

Reviewer Comments to Author:

The authors characterise the distribution of H3K27me3 across B. rapa leaves and inflorescence tissues, alongside its importance for appropriate development and flowering through gene silencing. Overall, the data appears to be solid providing a resource for the community and extends known relationships into new species. I highly commend the authors on the development and execution of the REA pipeline (aligning with FAIR principles), and hope this helps to establish a new "precedent" for increasing accessibility and rigour in our data & analyses.

I have some concerns/requests, which I think will strengthen the paper prior to publication:

1. While this study characterises the distribution of H3K27me3, it is not exploring anything epigenetic. Instead, this reference to epigenetics is reinforcing the vague use of the term, which is what the authors in ref 1 argue against. I recommend the first sentence should be deleted and all references to epigenetic(s) should instead refer to "histone modification(s)", "chromatin mark(s)" or "epigenome", where the latter is clearly defined.
2. Could the first use of "BraA.CLF" include the full name (i.e. define the abbreviation).
3. Could the authors please clarify whether "from the same plant samples" (p. 7) means from the same tissues but independent plants or aliquots of the same harvested tissue?
4. P. 8 Please include an appropriate citation for the sentence starting with "A metagene plot of H3K27me3 ... as described in other plant species, ..." for clarity.
5. For Fig 2A, can the signal from leaf vs inflorescence tissue be plotted separately? Could some measure of variance (standard deviation or standard error) be included in the plots?
6. Fig 2C, can the authors please mark the median. I find this more informative than the mean for this type of data.
7. P. 9, I am a bit concerned about the one-fold increase threshold used for ChIP signal. This threshold seems too low to reduce background noise and the analysis may benefit from using a threshold FC>1.2 - 1.5x (i.e. 20-50% increase from background).
8. Could the authors specify "H3K27me3" throughout manuscript. There are some references to "H3K27" or "H3K27 methylation", which should be replaced.
9. Could the authors please make Fig S2 more presentable. This could probably be combined with Fig S1.
10. Fig 4C - If high/med/low expression levels were based on inflorescence tissue, I would have expected more "lowly" expressed genes in the explored quadrant (down-regulated genes with increased H3K27me3). Instead there are many med-highly expressed genes in the "down-regulated" portion of the figure . There are also a number of "no_expr" in the up-regulated gene set. I am unsure how to reconcile this except to ask the authors to reproduce this figure/analysis (e.g. is it possible up- and down-regulated genes were switched or the contrast performed was relative to leaf instead of inflorescence?).

The p.adj-cutoff used here also seems higher than the commonly, yet arbitrary, used levels of 0.05 or 0.01. I would also appreciate any comments from the authors on this.

11. Can the authors please clarify the 1,724 overlap of genes with changed H3K27me3 and mRNA levels. Were the 4,729 deferential H3K27me3 genes overlapped with 13,377 DEGs to give 1,724 genes? Please also perform a Fisher's exact test for some level of statistical confidence.
12. P.12 sentence beginning with "All these developmental abnormalities ..." - please include citation for clarity.
13. A browser shot for H3K27me3 and mRNA at the B. rapa AG loci would be nice.
14. Please specify ChIP qPCR p. 12 sentence beginning with "We performed ChIP experiments...".
15. An ANOVA with post-hoc tukey tests should be performed for Fig 6 B-C.
16. Were braA.clf-1 seeds ensured to be homozygous?
17. Was RNA integrity checked prior to qPCR and 3' RNA sequencing?
18. If possible, could the authors re-analyse their raw qPCR fluorescence data using LinReg PCR (Ramakers et al 2003, Ruijter et al 2009).
19. P. 17, "A first step of trimming was performed with [52] v0.36.5 ." should be "A first step of trimming was performed with Trimmomatic (v0.36.5) [52].
20. Fig S4: could the authors please clarify how many flowers were tested for WT and clf-1?

Methods

Are the methods appropriate to the aims of the study, are they well described, and are necessary controls included? Choose an item.

Conclusions

Are the conclusions adequately supported by the data shown? Choose an item.

Reporting Standards

Does the manuscript adhere to the journal's guidelines on minimum standards of reporting? Choose an item.

Choose an item.

Statistics

Are you able to assess all statistics in the manuscript, including the appropriateness of statistical tests used? Choose an item.

Quality of Written English

Please indicate the quality of language in the manuscript: Choose an item.

Declaration of Competing Interests

Please complete a declaration of competing interests, considering the following questions:

- Have you in the past five years received reimbursements, fees, funding, or salary from an organisation that may in any way gain or lose financially from the publication of this manuscript, either now or in the future?
- Do you hold any stocks or shares in an organisation that may in any way gain or lose financially from the publication of this manuscript, either now or in the future?
- Do you hold or are you currently applying for any patents relating to the content of the manuscript?
- Have you received reimbursements, fees, funding, or salary from an organization that holds or has applied for patents relating to the content of the manuscript?
- Do you have any other financial competing interests?
- Do you have any non-financial competing interests in relation to this paper?

If you can answer no to all of the above, write 'I declare that I have no competing interests' below. If your reply is yes to any, please give details below.

I declare that I have no competing interests

I agree to the open peer review policy of the journal. I understand that my name will be included on my report to the authors and, if the manuscript is accepted for publication, my named report including any attachments I upload will be posted on the website along with the authors' responses. I agree for my report to be made available under an Open Access Creative Commons CC-BY license (http://creativecommons.org/licenses/by/4.0/). I understand that any comments which I do not wish to be included in my named report can be included as confidential comments to the editors, which will not be published.

Choose an item.

To further support our reviewers, we have joined with Publons, where you can gain additional credit to further highlight your hard work (see: https://publons.com/journal/530/gigascience). On publication of this paper, your review will be automatically added to Publons, you can then choose whether or not to claim your Publons credit. I understand this statement.

Yes Choose an item.
