# Supplementary material for: Genome-wide analysis of the H3K27me3 epigenome and transcriptome in Brassica rapa
Source: Gigascience. 2019 Dec 4;8(12):giz147. doi: 10.1093/gigascience/giz147 (PMC6892454; doi:10.1093/gigascience/giz147)
Supplement: giz147_Reviewer_2_Report_Original_Submission — Jingyu Zhang -- 9/1/2019 Reviewed [file giz147_reviewer_2_report_original_submission.pdf]

Reviewer Report

Title: Genome-wide analysis of the H3K27me3 epigenome and transcriptome in Brassica rapa

Version: Original Submission **Date:** 9/1/2019

Reviewer name: Jingyu Zhang

Reviewer Comments to Author:

In this MS, the author demonstrates the profile of H3K27me3 in different organ of Brassica rapa, and uncover its role in plant development with mutant analysis. Though the analysis of H3K27me3 has been done in B. rapa before, it focused only on specific region (Genes Genet Syst. 2016, 91:1-10). This study provides a genomic-wide view of this type of histone modification, enable us to get a comprehensive understanding of its function.

There are also some suggestions listed as below:

1. For high-throughput data, three independent biological repeats are required. Although the author demonstrates the reproducibility of the Epigenomic Analysis pipeline, please make it clear whether the replicate tests has been performed to ensure the accuracy of the results.
2. The H3K27me3 modification analysis has been done in both leaves and inflorescences. The Chip-seq data for inflorescences also deserves a figure to show its details.
3. For figure 5, the mutant and the wild type are in different pictures with different bars. If they are in the same picture, it would be much easier for the comparison. At least their bars should be the same.

Methods

Are the methods appropriate to the aims of the study, are they well described, and are necessary controls included? Choose an item.

Conclusions

Are the conclusions adequately supported by the data shown? Choose an item.

Reporting Standards

Does the manuscript adhere to the journal's guidelines on [minimum standards of reporting?]() Choose an item.

Choose an item.

Statistics

Are you able to assess all statistics in the manuscript, including the appropriateness of statistical tests used? Choose an item.

Quality of Written English

Please indicate the quality of language in the manuscript: Choose an item.

Declaration of Competing Interests

Please complete a declaration of competing interests, considering the following questions:

- Have you in the past five years received reimbursements, fees, funding, or salary from an organisation that may in any way gain or lose financially from the publication of this manuscript, either now or in the future?
- Do you hold any stocks or shares in an organisation that may in any way gain or lose financially from the publication of this manuscript, either now or in the future?
- Do you hold or are you currently applying for any patents relating to the content of the manuscript?
- Have you received reimbursements, fees, funding, or salary from an organization that holds or has applied for patents relating to the content of the manuscript?
- Do you have any other financial competing interests?
- Do you have any non-financial competing interests in relation to this paper?

If you can answer no to all of the above, write 'I declare that I have no competing interests' below. If your reply is yes to any, please give details below.

I declare that I have no competing interests

I agree to the open peer review policy of the journal. I understand that my name will be included on my report to the authors and, if the manuscript is accepted for publication, my named report including any attachments I upload will be posted on the website along with the authors' responses. I agree for my report to be made available under an Open Access Creative Commons CC-BY license (http://creativecommons.org/licenses/by/4.0/). I understand that any comments which I do not wish to be included in my named report can be included as confidential comments to the editors, which will not be published.

Choose an item.

To further support our reviewers, we have joined with Publons, where you can gain additional credit to further highlight your hard work (see: https://publons.com/journal/530/gigascience). On publication of this paper, your review will be automatically added to Publons, you can then choose whether or not to claim your Publons credit. I understand this statement.

Yes Choose an item.
